# Supplementary material for: DPWSS: differentially private working set selection for training support vector machines
Source: PeerJ Comput Sci. 2021 Dec 1;7:e799. doi: 10.7717/peerj-cs.799 (PMC8670395; doi:10.7717/peerj-cs.799)
Supplement: Supplemental Information 1 [file peerj-cs-07-799-s001.zip › CODE/matlab/Table/Table1.docx]

| Symbol | Description |
| --- | --- |
| *U* | Universe |
| *D*⊂*U* | Dataset to be trained |
| *D’* | Neighbor dataset of *D* |
| *x^i^__R^d^* | Train instance |
| *y^i^__*{1,-1} | Label of train instance |
| *α* | Dual variable |
| *e* | Vector of all ones |
| *C* | Upper bound of all variables |
| *K* | Kernel function |
| *Q* | Symmetric matrix of kernel function |
| *B* | Working set |
| *τ* | A small positive number |
| *σ* | Constant-factor |
| *M* | Mechanism |
| *Lap*(*λ*) | Laplace distribution with mean 0 and scale factor *λ* |
| *ɛ* | Privacy budget |
| *f* | Query function |
| *q*(*D*, *r*) | Score function |
| Δ*f* ,Δ*q* | Sensitivity of function |
| *TP* | True positive |
| *TN* | True negative |
| *FP* | False positive |
| *FN* | False negative |
