# Supplementary material for: DPWSS: differentially private working set selection for training support vector machines
Source: PeerJ Comput Sci. 2021 Dec 1;7:e799. doi: 10.7717/peerj-cs.799 (PMC8670395; doi:10.7717/peerj-cs.799)
Supplement: Supplemental Information 1 [file peerj-cs-07-799-s001.zip › CODE/matlab/Table/Table2.docx]

| Index | Dataset | #data | Range | #features | Imbalance ratio |
| --- | --- | --- | --- | --- | --- |
| 1 | a1a | 1605 | [0,1] | 119 | 0.33 |
| 2 | a5a | 6414 | [0,1] | 122 | 0.32 |
| 3 | australian | 690 | [-1,1] | 14 | 0.8 |
| 4 | breast | 683 | [-1,1] | 10 | 1.86 |
| 5 | diabetes | 768 | [-1,1] | 8 | 1.87 |
| 6 | fourclass | 862 | [-1,1] | 2 | 0.55 |
| 7 | german | 1000 | [-1,1] | 24 | 0.43 |
| 8 | gisette | 6000 | [-1,1] | 5000 | 1 |
| 9 | heart | 270 | [-1,1] | 13 | 0.8 |
| 10 | ijcnn1 | 49990 | [-1,1] | 22 | 0.11 |
| 11 | ionosphere | 351 | [-1,1] | 34 | 1.79 |
| 12 | rcv1 | 20242 | [-1,1] | 47236 | 1.08 |
| 13 | sonar | 208 | [-1,1] | 60 | 0.87 |
| 14 | splice | 1000 | [-1,1] | 60 | 1.07 |
| 15 | w1a | 2477 | [0,1] | 300 | 0.03 |
| 16 | w5a | 9888 | [0,1] | 300 | 0.03 |
