# Supplementary material for: DPWSS: differentially private working set selection for training support vector machines
Source: PeerJ Comput Sci. 2021 Dec 1;7:e799. doi: 10.7717/peerj-cs.799 (PMC8670395; doi:10.7717/peerj-cs.799)
Supplement: Supplemental Information 1 [file peerj-cs-07-799-s001.zip › CODE/matlab/Table/Table3.docx]

| dataset | shrinking | metrics | WSS2 | DPWSS | | | | | |
| --- | --- | --- | --- | --- | --- | --- | --- | --- | --- |
|  |  |  |  | epsi=1 sigm=0.1 | epsi=1 sigm=0.3 | epsi=1 sigm=0.5 | epsi=1 sigm=0.7 | epsi=0.5 sigm=0.7 | epsi=0.1 sigm=0.7 |
| a1a | 1 | AUC | 0.9117 | 0.9119 | 0.9117 | 0.9113 | 0.9116 | 0.9109 | 0.9123 |
|  |  | Accuracy | 0.8623 | 0.8611 | 0.8629 | 0.8629 | 0.8592 | 0.8604 | 0.8636 |
|  |  | Precision | 0.7669 | 0.7654 | 0.7709 | 0.7709 | 0.76 | 0.7631 | 0.7667 |
|  |  | Recall | 0.6329 | 0.6278 | 0.6304 | 0.6304 | 0.6253 | 0.6278 | 0.6405 |
|  |  | F1 | 0.6935 | 0.6898 | 0.6936 | 0.6936 | 0.6861 | 0.6889 | 0.6979 |
|  |  | Mcc | 0.6104 | 0.6063 | 0.6115 | 0.6115 | 0.6012 | 0.6048 | 0.6148 |
|  |  | obj | -540.57 | -540.42 | -540.45 | -540.42 | -539.95 | -540.23 | -540.06 |
|  |  | iteration | 8649 | 10535 | 6735 | 4316 | 3239 | 3529 | 3406 |
|  | 0 | AUC | 0.9117 | 0.9116 | 0.9116 | 0.9119 | 0.9122 | 0.9111 | 0.9117 |
|  |  | Accuracy | 0.8623 | 0.8623 | 0.8629 | 0.8629 | 0.8617 | 0.8623 | 0.8617 |
|  |  | Precision | 0.7669 | 0.7685 | 0.7692 | 0.7709 | 0.7645 | 0.7685 | 0.7645 |
|  |  | Recall | 0.6329 | 0.6304 | 0.6329 | 0.6304 | 0.6329 | 0.6304 | 0.6329 |
|  |  | F1 | 0.6935 | 0.6926 | 0.6944 | 0.6936 | 0.6925 | 0.6926 | 0.6925 |
|  |  | Mcc | 0.6104 | 0.61 | 0.612 | 0.6115 | 0.6088 | 0.61 | 0.6088 |
|  |  | obj | -540.57 | -540.42 | -540.44 | -540.38 | -540.12 | -540.11 | -540.2 |
|  |  | iteration | 7997 | 9566 | 6091 | 4252 | 3447 | 3379 | 3295 |
| a5a | 1 | AUC | 0.906 | 0.9059 | 0.9059 | 0.9057 | 0.9058 | 0.9058 | 0.9059 |
|  |  | Accuracy | 0.8506 | 0.8502 | 0.8499 | 0.8511 | 0.8486 | 0.8506 | 0.8503 |
|  |  | Precision | 0.7327 | 0.7317 | 0.7306 | 0.7354 | 0.7311 | 0.7334 | 0.7305 |
|  |  | Recall | 0.6131 | 0.6119 | 0.6119 | 0.6112 | 0.6029 | 0.6119 | 0.615 |
|  |  | F1 | 0.6676 | 0.6664 | 0.666 | 0.6676 | 0.6608 | 0.6671 | 0.6678 |
|  |  | Mcc | 0.576 | 0.5746 | 0.5738 | 0.5768 | 0.5689 | 0.5758 | 0.5757 |
|  |  | obj | -2224.72 | -2224.56 | -2224.57 | -2224.33 | -2223.2 | -2223.75 | -2223.92 |
|  |  | iteration | 35752 | 36151 | 22590 | 15987 | 13240 | 14034 | 14275 |
|  | 0 | AUC | 0.906 | 0.9058 | 0.9059 | 0.9058 | 0.9057 | 0.9058 | 0.906 |
|  |  | Accuracy | 0.8506 | 0.8506 | 0.8506 | 0.8514 | 0.8503 | 0.8502 | 0.85 |
|  |  | Precision | 0.7327 | 0.7323 | 0.7327 | 0.7373 | 0.7312 | 0.7331 | 0.7322 |
|  |  | Recall | 0.6131 | 0.6138 | 0.6131 | 0.6099 | 0.6138 | 0.6093 | 0.6099 |
|  |  | F1 | 0.6676 | 0.6678 | 0.6676 | 0.6676 | 0.6674 | 0.6655 | 0.6655 |
|  |  | Mcc | 0.576 | 0.5762 | 0.576 | 0.5773 | 0.5754 | 0.5741 | 0.5738 |
|  |  | obj | -2224.72 | -2224.41 | -2224.47 | -2224.33 | -2223.72 | -2223.07 | -2223.85 |
|  |  | iteration | 37578 | 33682 | 21592 | 16418 | 13926 | 12987 | 14318 |
| australian | 1 | AUC | 0.9393 | 0.9403 | 0.9378 | 0.9318 | 0.9141 | 0.9126 | 0.9202 |
|  |  | Accuracy | 0.8565 | 0.8565 | 0.8565 | 0.8565 | 0.8565 | 0.8565 | 0.8565 |
|  |  | Precision | 0.7873 | 0.7873 | 0.7873 | 0.7873 | 0.7873 | 0.7873 | 0.7873 |
|  |  | Recall | 0.9283 | 0.9283 | 0.9283 | 0.9283 | 0.9283 | 0.9283 | 0.9283 |
|  |  | F1 | 0.852 | 0.852 | 0.852 | 0.852 | 0.852 | 0.852 | 0.852 |
|  |  | Mcc | 0.7237 | 0.7237 | 0.7237 | 0.7237 | 0.7237 | 0.7237 | 0.7237 |
|  |  | obj | -199.65 | -199.25 | -198.98 | -198.21 | -197.78 | -198.53 | -198.64 |
|  |  | iteration | 10727 | 6438 | 1910 | 835 | 493 | 596 | 612 |
|  | 0 | AUC | 0.9393 | 0.9397 | 0.9324 | 0.9111 | 0.923 | 0.9223 | 0.9316 |
|  |  | Accuracy | 0.8565 | 0.8565 | 0.8565 | 0.8565 | 0.8565 | 0.8565 | 0.8565 |
|  |  | Precision | 0.7873 | 0.7873 | 0.7873 | 0.7873 | 0.7873 | 0.7873 | 0.7873 |
|  |  | Recall | 0.9283 | 0.9283 | 0.9283 | 0.9283 | 0.9283 | 0.9283 | 0.9283 |
|  |  | F1 | 0.852 | 0.852 | 0.852 | 0.852 | 0.852 | 0.852 | 0.852 |
|  |  | Mcc | 0.7237 | 0.7237 | 0.7237 | 0.7237 | 0.7237 | 0.7237 | 0.7237 |
|  |  | obj | -199.65 | -199.25 | -199.15 | -198.68 | -198.33 | -198.62 | -198.82 |
|  |  | iteration | 10590 | 6978 | 2629 | 847 | 542 | 637 | 731 |
| breast | 1 | AUC | 0.9962 | 0.9962 | 0.9963 | 0.9961 | 0.9962 | 0.9961 | 0.9962 |
|  |  | Accuracy | 0.9707 | 0.9707 | 0.9707 | 0.9707 | 0.9707 | 0.9707 | 0.9707 |
|  |  | Precision | 0.9818 | 0.9818 | 0.9818 | 0.9818 | 0.9818 | 0.9818 | 0.9818 |
|  |  | Recall | 0.973 | 0.973 | 0.973 | 0.973 | 0.973 | 0.973 | 0.973 |
|  |  | F1 | 0.9774 | 0.9774 | 0.9774 | 0.9774 | 0.9774 | 0.9774 | 0.9774 |
|  |  | Mcc | 0.936 | 0.936 | 0.936 | 0.936 | 0.936 | 0.936 | 0.936 |
|  |  | obj | -46 | -45.96 | -45.93 | -45.89 | -45.63 | -45.53 | -45.78 |
|  |  | iteration | 212 | 542 | 257 | 196 | 138 | 146 | 150 |
|  | 0 | AUC | 0.9962 | 0.9962 | 0.9963 | 0.9963 | 0.9962 | 0.9962 | 0.9962 |
|  |  | Accuracy | 0.9707 | 0.9707 | 0.9707 | 0.9707 | 0.9722 | 0.9722 | 0.9722 |
|  |  | Precision | 0.9818 | 0.9818 | 0.9818 | 0.9818 | 0.9819 | 0.9841 | 0.9819 |
|  |  | Recall | 0.973 | 0.973 | 0.973 | 0.973 | 0.9752 | 0.973 | 0.9752 |
|  |  | F1 | 0.9774 | 0.9774 | 0.9774 | 0.9774 | 0.9785 | 0.9785 | 0.9785 |
|  |  | Mcc | 0.936 | 0.936 | 0.936 | 0.936 | 0.9391 | 0.9393 | 0.9391 |
|  |  | obj | -46 | -45.95 | -45.99 | -45.78 | -45.62 | -45.87 | -45.88 |
|  |  | iteration | 212 | 443 | 329 | 184 | 146 | 160 | 181 |
| diabetes | 1 | AUC | 0.8388 | 0.8393 | 0.839 | 0.8388 | 0.8383 | 0.8378 | 0.8385 |
|  |  | Accuracy | 0.776 | 0.7747 | 0.7734 | 0.7747 | 0.7708 | 0.7721 | 0.7721 |
|  |  | Precision | 0.7918 | 0.7904 | 0.789 | 0.7904 | 0.7893 | 0.7886 | 0.7897 |
|  |  | Recall | 0.89 | 0.89 | 0.89 | 0.89 | 0.884 | 0.888 | 0.886 |
|  |  | F1 | 0.838 | 0.8373 | 0.8365 | 0.8373 | 0.834 | 0.8354 | 0.8351 |
|  |  | Mcc | 0.4878 | 0.4846 | 0.4813 | 0.4846 | 0.4759 | 0.4784 | 0.4788 |
|  |  | obj | -403.1 | -403.03 | -402.97 | -403 | -402.53 | -402.44 | -402.58 |
|  |  | iteration | 680 | 873 | 612 | 590 | 460 | 475 | 502 |
|  | 0 | AUC | 0.8388 | 0.8392 | 0.8391 | 0.8393 | 0.8384 | 0.8383 | 0.839 |
|  |  | Accuracy | 0.776 | 0.7747 | 0.776 | 0.7747 | 0.7695 | 0.7721 | 0.776 |
|  |  | Precision | 0.7918 | 0.7904 | 0.7918 | 0.7914 | 0.7858 | 0.7897 | 0.7918 |
|  |  | Recall | 0.89 | 0.89 | 0.89 | 0.888 | 0.888 | 0.886 | 0.89 |
|  |  | F1 | 0.838 | 0.8373 | 0.838 | 0.8369 | 0.8338 | 0.8351 | 0.838 |
|  |  | Mcc | 0.4878 | 0.4846 | 0.4878 | 0.48449 | 0.4718 | 0.4788 | 0.4878 |
|  |  | obj | -403.1 | -403 | -403.03 | -403 | -402.05 | -402.8 | -402.72 |
|  |  | iteration | 680 | 793 | 687 | 529 | 490 | 502 | 516 |
| fourclass | 1 | AUC | 0.8266 | 0.8268 | 0.8262 | 0.8265 | 0.8255 | 0.8251 | 0.8261 |
|  |  | Accuracy | 0.7715 | 0.7715 | 0.7715 | 0.7715 | 0.7715 | 0.7726 | 0.7726 |
|  |  | Precision | 0.7455 | 0.7455 | 0.7455 | 0.7477 | 0.7477 | 0.7489 | 0.7489 |
|  |  | Recall | 0.544 | 0.544 | 0.544 | 0.5407 | 0.5407 | 0.544 | 0.544 |
|  |  | F1 | 0.629 | 0.629 | 0.629 | 0.6276 | 0.6276 | 0.6302 | 0.6302 |
|  |  | Mcc | 0.4818 | 0.4818 | 0.4818 | 0.4816 | 0.4816 | 0.4845 | 0.4845 |
|  |  | obj | -454.29 | -454.27 | -454.22 | -454.23 | -454.12 | -454.12 | -454.17 |
|  |  | iteration | 590 | 917 | 676 | 509 | 450 | 466 | 496 |
|  | 0 | AUC | 0.8266 | 0.827 | 0.8256 | 0.8272 | 0.8245 | 0.8263 | 0.8257 |
|  |  | Accuracy | 0.7715 | 0.7691 | 0.7715 | 0.7691 | 0.7749 | 0.7726 | 0.7726 |
|  |  | Precision | 0.7455 | 0.7432 | 0.7455 | 0.7432 | 0.7653 | 0.7534 | 0.7467 |
|  |  | Recall | 0.544 | 0.5375 | 0.544 | 0.5375 | 0.5309 | 0.5375 | 0.5472 |
|  |  | F1 | 0.629 | 0.6238 | 0.629 | 0.6238 | 0.6269 | 0.6274 | 0.6316 |
|  |  | Mcc | 0.4818 | 0.4761 | 0.4818 | 0.4761 | 0.4894 | 0.4842 | 0.4847 |
|  |  | obj | -454.29 | -454.25 | -454.22 | -454.18 | -453.68 | -453.8 | -454.19 |
|  |  | iteration | 590 | 908 | 625 | 526 | 421 | 458 | 471 |
| german | 1 | AUC | 0.8165 | 0.8163 | 0.8161 | 0.816 | 0.8161 | 0.8157 | 0.816 |
|  |  | Accuracy | 0.789 | 0.788 | 0.787 | 0.786 | 0.785 | 0.783 | 0.784 |
|  |  | Precision | 0.6943 | 0.6947 | 0.69 | 0.6886 | 0.6856 | 0.6861 | 0.6858 |
|  |  | Recall | 0.53 | 0.5233 | 0.5267 | 0.5233 | 0.5233 | 0.51 | 0.5167 |
|  |  | F1 | 0.6011 | 0.597 | 0.5974 | 0.5947 | 0.5936 | 0.5851 | 0.5894 |
|  |  | Mcc | 0.469 | 0.4654 | 0.4638 | 0.4608 | 0.4586 | 0.4514 | 0.455 |
|  |  | obj | -519.05 | -518.76 | -518.81 | -518.48 | -517.51 | -517.92 | -517.59 |
|  |  | iteration | 13688 | 9415 | 5821 | 4533 | 3675 | 3741 | 3787 |
|  | 0 | AUC | 0.8165 | 0.8163 | 0.8163 | 0.8155 | 0.8154 | 0.8159 | 0.8162 |
|  |  | Accuracy | 0.789 | 0.787 | 0.789 | 0.783 | 0.785 | 0.784 | 0.787 |
|  |  | Precision | 0.6943 | 0.6916 | 0.6978 | 0.6781 | 0.6856 | 0.6826 | 0.6933 |
|  |  | Recall | 0.53 | 0.5233 | 0.5233 | 0.5267 | 0.5233 | 0.5233 | 0.52 |
|  |  | F1 | 0.6011 | 0.5958 | 0.5981 | 0.5929 | 0.5936 | 0.5925 | 0.5943 |
|  |  | Mcc | 0.469 | 0.4631 | 0.4677 | 0.4548 | 0.4586 | 0.4563 | 0.4625 |
|  |  | obj | -519.05 | -518.64 | -518.69 | -518.41 | -517.91 | -517.98 | -517.65 |
|  |  | iteration | 13454 | 9367 | 5495 | 4576 | 3663 | 3842 | 3742 |
| gisette | 1 | AUC | 1 | 1 | 1 | 1 | 1 | 1 | 1 |
|  |  | Accuracy | 1 | 1 | 1 | 1 | 1 | 1 | 1 |
|  |  | Precision | 1 | 1 | 1 | 1 | 1 | 1 | 1 |
|  |  | Recall | 1 | 1 | 1 | 1 | 1 | 1 | 1 |
|  |  | F1 | 1 | 1 | 1 | 1 | 1 | 1 | 1 |
|  |  | Mcc | 1 | 1 | 1 | 1 | 1 | 1 | 1 |
|  |  | obj | -0.668 | -0.668 | -0.668 | -0.668 | -0.668 | -0.668 | -0.668 |
|  |  | iteration | 8157 | 23247 | 9312 | 6736 | 6002 | 5978 | 5979 |
|  | 0 | AUC | 1 | 1 | 1 | 1 | 1 | 1 | 1 |
|  |  | Accuracy | 1 | 1 | 1 | 1 | 1 | 1 | 1 |
|  |  | Precision | 1 | 1 | 1 | 1 | 1 | 1 | 1 |
|  |  | Recall | 1 | 1 | 1 | 1 | 1 | 1 | 1 |
|  |  | F1 | 1 | 1 | 1 | 1 | 1 | 1 | 1 |
|  |  | Mcc | 1 | 1 | 1 | 1 | 1 | 1 | 1 |
|  |  | obj | -0.668 | -0.668 | -0.668 | -0.668 | -0.668 | -0.668 | -0.668 |
|  |  | iteration | 8246 | 17902 | 7933 | 6918 | 6258 | 6225 | 6250 |
| heart | 1 | AUC | 0.9282 | 0.9281 | 0.9287 | 0.9296 | 0.9287 | 0.9282 | 0.9296 |
|  |  | Accuracy | 0.8481 | 0.8444 | 0.8481 | 0.8481 | 0.8519 | 0.8593 | 0.8481 |
|  |  | Precision | 0.8376 | 0.8362 | 0.8376 | 0.8376 | 0.8509 | 0.8534 | 0.8376 |
|  |  | Recall | 0.8167 | 0.8083 | 0.8167 | 0.8167 | 0.8083 | 0.825 | 0.8167 |
|  |  | F1 | 0.827 | 0.822 | 0.827 | 0.827 | 0.8291 | 0.839 | 0.827 |
|  |  | Mcc | 0.6919 | 0.6843 | 0.6919 | 0.6919 | 0.6992 | 0.7144 | 0.6919 |
|  |  | obj | -92.47 | -92.07 | -92.33 | -92.17 | -90.78 | -91.34 | -91.62 |
|  |  | iteration | 1010 | 992 | 662 | 525 | 372 | 404 | 410 |
|  | 0 | AUC | 0.9282 | 0.9278 | 0.9284 | 0.9292 | 0.9251 | 0.9278 | 0.9275 |
|  |  | Accuracy | 0.8481 | 0.8481 | 0.8444 | 0.8556 | 0.8556 | 0.8593 | 0.8481 |
|  |  | Precision | 0.8376 | 0.8435 | 0.8362 | 0.8462 | 0.8584 | 0.8596 | 0.8376 |
|  |  | Recall | 0.8167 | 0.8083 | 0.8083 | 0.825 | 0.8083 | 0.8167 | 0.8167 |
|  |  | F1 | 0.827 | 0.8255 | 0.822 | 0.8354 | 0.8326 | 0.8376 | 0.827 |
|  |  | Mcc | 0.6919 | 0.6917 | 0.6843 | 0.7069 | 0.7068 | 0.7143 | 0.6919 |
|  |  | obj | -92.47 | -92.2 | -92.07 | -92.09 | -91.03 | -91.14 | -91.36 |
|  |  | iteration | 1010 | 1097 | 671 | 542 | 380 | 390 | 411 |
| ijcnn1 | 1 | AUC | 0.918 | 0.918 | 0.918 | 0.9179 | 0.917 | 0.9184 | 0.9179 |
|  |  | Accuracy | 0.9242 | 0.9242 | 0.9241 | 0.9242 | 0.9241 | 0.9241 | 0.9241 |
|  |  | Precision | 0.7579 | 0.758 | 0.7576 | 0.7581 | 0.767 | 0.7565 | 0.7598 |
|  |  | Recall | 0.3219 | 0.3215 | 0.3215 | 0.3217 | 0.314 | 0.3221 | 0.3188 |
|  |  | F1 | 0.4518 | 0.4515 | 0.4514 | 0.4517 | 0.4456 | 0.4518 | 0.4491 |
|  |  | Mcc | 0.4628 | 0.4626 | 0.4624 | 0.4628 | 0.4604 | 0.4625 | 0.4612 |
|  |  | obj | -8590.16 | -8590.07 | -8590.11 | -8590.07 | -8588.99 | -8588.82 | -8589 |
|  |  | iteration | 18382 | 40443 | 29635 | 25599 | 19150 | 18694 | 17842 |
|  | 0 | AUC | 0.918 | 0.9181 | 0.918 | 0.9181 | 0.9181 | 0.9183 | 0.9183 |
|  |  | Accuracy | 0.9241 | 0.924 | 0.9241 | 0.9241 | 0.9238 | 0.9242 | 0.924 |
|  |  | Precision | 0.7574 | 0.7573 | 0.7573 | 0.7572 | 0.7549 | 0.7569 | 0.7562 |
|  |  | Recall | 0.3217 | 0.3202 | 0.3215 | 0.3212 | 0.3179 | 0.3221 | 0.3208 |
|  |  | F1 | 0.4515 | 0.4501 | 0.4513 | 0.4511 | 0.4474 | 0.4519 | 0.4505 |
|  |  | Mcc | 0.4625 | 0.4614 | 0.4623 | 0.4621 | 0.4588 | 0.4626 | 0.4614 |
|  |  | obj | -8590.16 | -8590.05 | -8590.1 | -8589.94 | -8588.84 | -8589.73 | -8589.18 |
|  |  | iteration | 16469 | 45191 | 30133 | 23786 | 18416 | 20637 | 18750 |
| ionosphere | 1 | AUC | 0.9677 | 0.9684 | 0.9681 | 0.9679 | 0.9686 | 0.9688 | 0.9687 |
|  |  | Accuracy | 0.9373 | 0.9373 | 0.9373 | 0.9259 | 0.9345 | 0.9373 | 0.9345 |
|  |  | Precision | 0.9283 | 0.9283 | 0.9283 | 0.9234 | 0.9244 | 0.9356 | 0.9316 |
|  |  | Recall | 0.9778 | 0.9778 | 0.9778 | 0.9644 | 0.9778 | 0.9689 | 0.9689 |
|  |  | F1 | 0.9524 | 0.9524 | 0.9524 | 0.9435 | 0.9503 | 0.952 | 0.9499 |
|  |  | Mcc | 0.8634 | 0.8634 | 0.8634 | 0.8379 | 0.8572 | 0.863 | 0.8567 |
|  |  | obj | -73.41 | -73.41 | -73.31 | -71.97 | -72.02 | -72.44 | -72.11 |
|  |  | iteration | 1016 | 1489 | 834 | 664 | 555 | 562 | 525 |
|  | 0 | AUC | 0.9677 | 0.9674 | 0.9678 | 0.9673 | 0.9651 | 0.9667 | 0.965 |
|  |  | Accuracy | 0.9373 | 0.9288 | 0.9373 | 0.9288 | 0.9316 | 0.9288 | 0.9288 |
|  |  | Precision | 0.9283 | 0.9274 | 0.9283 | 0.9237 | 0.9277 | 0.9237 | 0.9274 |
|  |  | Recall | 0.9778 | 0.9644 | 0.9778 | 0.9689 | 0.9689 | 0.9689 | 0.9644 |
|  |  | F1 | 0.9524 | 0.9455 | 0.9524 | 0.9458 | 0.9478 | 0.9458 | 0.9455 |
|  |  | Mcc | 0.8634 | 0.8441 | 0.8634 | 0.8442 | 0.8505 | 0.8442 | 0.8441 |
|  |  | obj | -73.41 | -73.15 | -73.2 | -73.13 | -72.44 | -72.85 | -71.91 |
|  |  | iteration | 770 | 1348 | 944 | 761 | 560 | 610 | 548 |
| rcv1 | 1 | AUC | 0.9989 | 0.9989 | 0.9989 | 0.9989 | 0.9989 | 0.9989 | 0.9989 |
|  |  | Accuracy | 0.9896 | 0.9896 | 0.9896 | 0.9896 | 0.9896 | 0.9896 | 0.9896 |
|  |  | Precision | 0.9896 | 0.9896 | 0.9896 | 0.9896 | 0.9896 | 0.9896 | 0.9897 |
|  |  | Recall | 0.9903 | 0.9903 | 0.9903 | 0.9903 | 0.9903 | 0.9903 | 0.9903 |
|  |  | F1 | 0.9899 | 0.9899 | 0.9899 | 0.9899 | 0.9899 | 0.9899 | 0.99 |
|  |  | Mcc | 0.9791 | 0.9791 | 0.9791 | 0.9791 | 0.9791 | 0.9791 | 0.9792 |
|  |  | obj | -1745.67 | -1745.66 | -1745.65 | -1745.62 | -1745.63 | -1745.6 | -1745.59 |
|  |  | iteration | 11639 | 41681 | 17129 | 12374 | 11029 | 9865 | 9945 |
|  | 0 | AUC | 0.9989 | 0.9989 | 0.9989 | 0.9989 | 0.9989 | 0.9989 | 0.9989 |
|  |  | Accuracy | 0.9896 | 0.9896 | 0.9896 | 0.9896 | 0.9896 | 0.9896 | 0.9896 |
|  |  | Precision | 0.9896 | 0.9896 | 0.9896 | 0.9896 | 0.9896 | 0.9897 | 0.9896 |
|  |  | Recall | 0.9903 | 0.9903 | 0.9903 | 0.9903 | 0.9903 | 0.9903 | 0.9903 |
|  |  | F1 | 0.9899 | 0.9899 | 0.9899 | 0.9899 | 0.9899 | 0.99 | 0.9899 |
|  |  | Mcc | 0.9791 | 0.9791 | 0.9791 | 0.9791 | 0.9791 | 0.9792 | 0.9791 |
|  |  | obj | -1745.67 | -1745.66 | -1745.65 | -1745.64 | -1745.55 | -1745.58 | -1745.61 |
|  |  | iteration | 11650 | 38114 | 16388 | 13014 | 9242 | 9419 | 10457 |
| sonar | 1 | AUC | 0.9495 | 0.9491 | 0.9475 | 0.9467 | 0.9489 | 0.9459 | 0.9482 |
|  |  | Accuracy | 0.8942 | 0.8894 | 0.8894 | 0.8894 | 0.8942 | 0.899 | 0.8894 |
|  |  | Precision | 0.8641 | 0.8558 | 0.8558 | 0.8558 | 0.8641 | 0.8654 | 0.8558 |
|  |  | Recall | 0.9175 | 0.9175 | 0.9175 | 0.9175 | 0.9175 | 0.9278 | 0.9175 |
|  |  | F1 | 0.89 | 0.8856 | 0.8856 | 0.8856 | 0.89 | 0.8955 | 0.8856 |
|  |  | Mcc | 0.7896 | 0.7806 | 0.7806 | 0.7806 | 0.7896 | 0.7999 | 0.7806 |
|  |  | obj | -65.67 | -65.62 | -65.48 | -65.49 | -65.21 | -64.79 | -65 |
|  |  | iteration | 1492 | 1716 | 1035 | 687 | 544 | 478 | 473 |
|  | 0 | AUC | 0.9496 | 0.9501 | 0.9475 | 0.9464 | 0.9492 | 0.9455 | 0.9439 |
|  |  | Accuracy | 0.8942 | 0.8894 | 0.8894 | 0.8894 | 0.899 | 0.899 | 0.8846 |
|  |  | Precision | 0.8641 | 0.8558 | 0.8558 | 0.8558 | 0.8654 | 0.8585 | 0.8476 |
|  |  | Recall | 0.9175 | 0.9175 | 0.9175 | 0.9175 | 0.9278 | 0.9381 | 0.9175 |
|  |  | F1 | 0.89 | 0.8856 | 0.8856 | 0.8856 | 0.8955 | 0.8966 | 0.8812 |
|  |  | Mcc | 0.7896 | 0.7806 | 0.7806 | 0.7806 | 0.7999 | 0.8013 | 0.7717 |
|  |  | obj | -65.67 | -65.57 | -65.43 | -65.49 | -64.97 | -65.02 | -64.88 |
|  |  | iteration | 1397 | 1516 | 929 | 701 | 489 | 448 | 440 |
| splice | 1 | AUC | 0.9173 | 0.9165 | 0.9164 | 0.9169 | 0.9165 | 0.9173 | 0.9162 |
|  |  | Accuracy | 0.842 | 0.84 | 0.842 | 0.845 | 0.841 | 0.84 | 0.844 |
|  |  | Precision | 0.8671 | 0.8665 | 0.8716 | 0.8724 | 0.8698 | 0.868 | 0.8737 |
|  |  | Recall | 0.8201 | 0.8162 | 0.8143 | 0.8201 | 0.8143 | 0.8143 | 0.8162 |
|  |  | F1 | 0.8429 | 0.8406 | 0.842 | 0.8455 | 0.8412 | 0.8403 | 0.844 |
|  |  | Mcc | 0.6853 | 0.6815 | 0.6859 | 0.6916 | 0.6838 | 0.6817 | 0.69 |
|  |  | obj | -375.19 | -374.55 | -374.56 | -374.31 | -373.25 | -374.02 | -373.51 |
|  |  | iteration | 95972 | 19779 | 11627 | 8079 | 6486 | 6847 | 6620 |
|  | 0 | AUC | 0.9173 | 0.9164 | 0.9169 | 0.9156 | 0.9166 | 0.9163 | 0.9168 |
|  |  | Accuracy | 0.842 | 0.845 | 0.843 | 0.843 | 0.841 | 0.844 | 0.841 |
|  |  | Precision | 0.8671 | 0.8724 | 0.8719 | 0.8719 | 0.8698 | 0.8737 | 0.8698 |
|  |  | Recall | 0.8201 | 0.8201 | 0.8162 | 0.8162 | 0.8143 | 0.8162 | 0.8143 |
|  |  | F1 | 0.8429 | 0.8455 | 0.8432 | 0.8432 | 0.8412 | 0.844 | 0.8412 |
|  |  | Mcc | 0.6853 | 0.6916 | 0.6878 | 0.6878 | 0.6838 | 0.69 | 0.6838 |
|  |  | obj | -375.19 | -374.08 | -374.11 | -373.89 | -373.32 | -373.77 | -373.7 |
|  |  | iteration | 38987 | 18752 | 11058 | 7562 | 6501 | 6845 | 6785 |
| w1a | 1 | AUC | 0.9755 | 0.9759 | 0.9757 | 0.9757 | 0.9757 | 0.9756 | 0.9754 |
|  |  | Accuracy | 0.9927 | 0.9927 | 0.9927 | 0.9927 | 0.9927 | 0.9927 | 0.9927 |
|  |  | Precision | 0.9821 | 0.9821 | 0.9821 | 0.9821 | 0.9821 | 0.9821 | 0.9821 |
|  |  | Recall | 0.7639 | 0.7639 | 0.7639 | 0.7639 | 0.7639 | 0.7639 | 0.7639 |
|  |  | F1 | 0.8594 | 0.8594 | 0.8594 | 0.8594 | 0.8594 | 0.8594 | 0.8594 |
|  |  | Mcc | 0.8628 | 0.8628 | 0.8628 | 0.8628 | 0.8628 | 0.8628 | 0.8628 |
|  |  | obj | -62.92 | -62.89 | -62.91 | -62.9 | -62.85 | -62.89 | -62.89 |
|  |  | iteration | 2565 | 9030 | 5034 | 3529 | 1835 | 1949 | 2224 |
|  | 0 | AUC | 0.9755 | 0.9755 | 0.9758 | 0.9759 | 0.9766 | 0.9758 | 0.9734 |
|  |  | Accuracy | 0.9927 | 0.9927 | 0.9927 | 0.9927 | 0.9927 | 0.9927 | 0.9927 |
|  |  | Precision | 0.9821 | 0.9821 | 0.9821 | 0.9821 | 0.9821 | 0.9821 | 0.9821 |
|  |  | Recall | 0.7639 | 0.7639 | 0.7639 | 0.7639 | 0.7639 | 0.7639 | 0.7639 |
|  |  | F1 | 0.8594 | 0.8594 | 0.8594 | 0.8594 | 0.8594 | 0.8594 | 0.8594 |
|  |  | Mcc | 0.8628 | 0.8628 | 0.8628 | 0.8628 | 0.8628 | 0.8628 | 0.8628 |
|  |  | obj | -62.92 | -62.89 | -62.89 | -62.79 | -62.8 | -62.86 | -62.85 |
|  |  | iteration | 2547 | 8436 | 4326 | 2222 | 1691 | 1781 | 1737 |
| w5a | 1 | AUC | 0.9632 | 0.9632 | 0.9638 | 0.9632 | 0.9625 | 0.9653 | 0.9626 |
|  |  | Accuracy | 0.9889 | 0.9887 | 0.9888 | 0.9887 | 0.9887 | 0.9889 | 0.9887 |
|  |  | Precision | 0.9524 | 0.9424 | 0.9474 | 0.9424 | 0.9424 | 0.9524 | 0.9424 |
|  |  | Recall | 0.6406 | 0.6406 | 0.6406 | 0.6406 | 0.6406 | 0.6406 | 0.6406 |
|  |  | F1 | 0.766 | 0.7627 | 0.7643 | 0.7627 | 0.7627 | 0.766 | 0.7627 |
|  |  | Mcc | 0.7762 | 0.772 | 0.7741 | 0.772 | 0.772 | 0.7762 | 0.772 |
|  |  | obj | -291.68 | -291.55 | -291.6 | -291.6 | -291.37 | -290.15 | -291.29 |
|  |  | iteration | 15422 | 31105 | 13388 | 10568 | 6514 | 5906 | 5858 |
|  | 0 | AUC | 0.9632 | 0.9636 | 0.9632 | 0.9633 | 0.9632 | 0.9623 | 0.9626 |
|  |  | Accuracy | 0.9889 | 0.9888 | 0.9887 | 0.9888 | 0.9889 | 0.9888 | 0.9887 |
|  |  | Precision | 0.9524 | 0.9427 | 0.9424 | 0.9474 | 0.9476 | 0.9474 | 0.9424 |
|  |  | Recall | 0.6406 | 0.6441 | 0.6406 | 0.6406 | 0.6441 | 0.6406 | 0.6406 |
|  |  | F1 | 0.766 | 0.7653 | 0.7627 | 0.7643 | 0.7669 | 0.7643 | 0.7627 |
|  |  | Mcc | 0.7762 | 0.7743 | 0.772 | 0.7741 | 0.7764 | 0.7741 | 0.772 |
|  |  | obj | -291.68 | -291.53 | -291.55 | -291.46 | -291.39 | -291.4 | -291.31 |
|  |  | iteration | 15511 | 25969 | 13074 | 8879 | 6630 | 6373 | 5838 |
