# Supplementary figures and images for: DPWSS: differentially private working set selection for training support vector machines
Source: PeerJ Comput Sci. 2021 Dec 1;7:e799. doi: 10.7717/peerj-cs.799 (PMC8670395; doi:10.7717/peerj-cs.799)

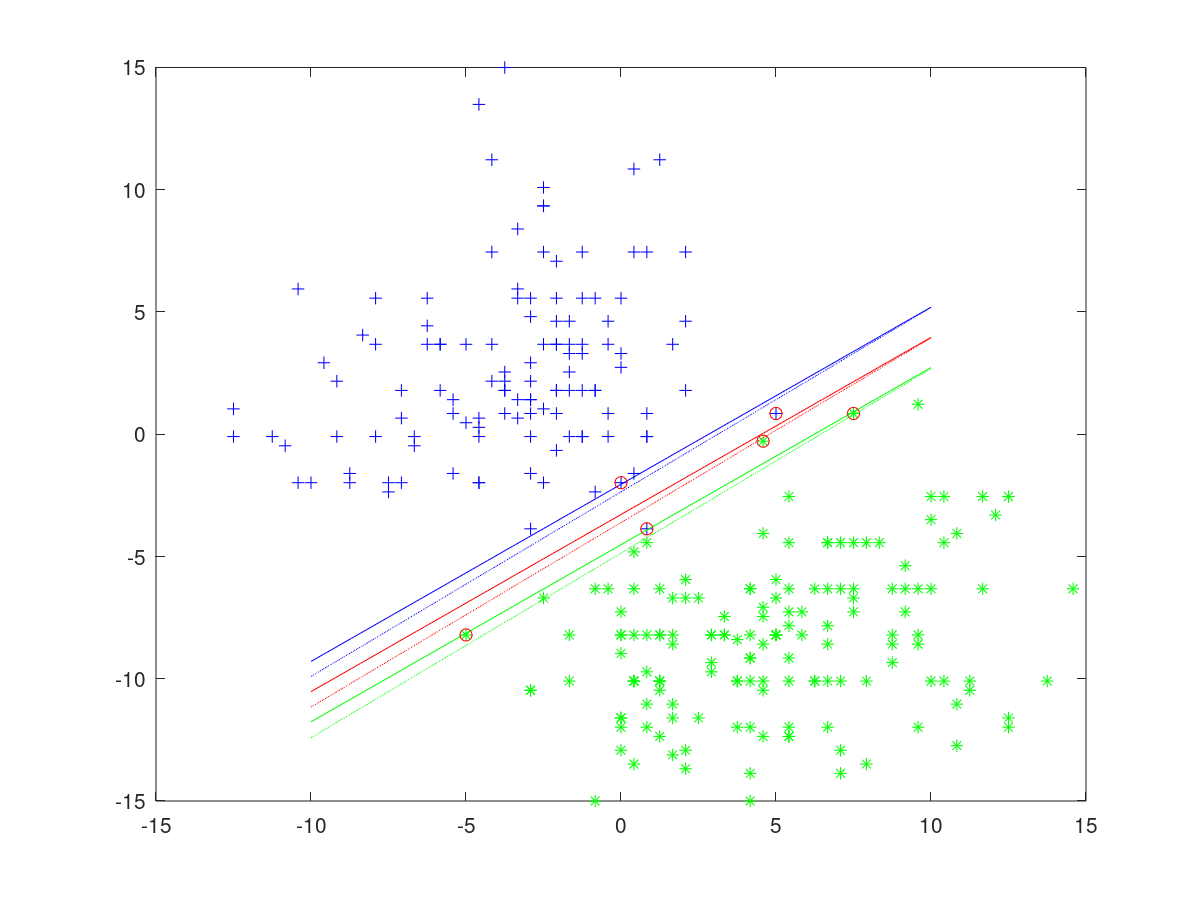

Supplement: Supplemental Information 1 [file peerj-cs-07-799-s001.zip › CODE/matlab/Figure/Figure1.png]

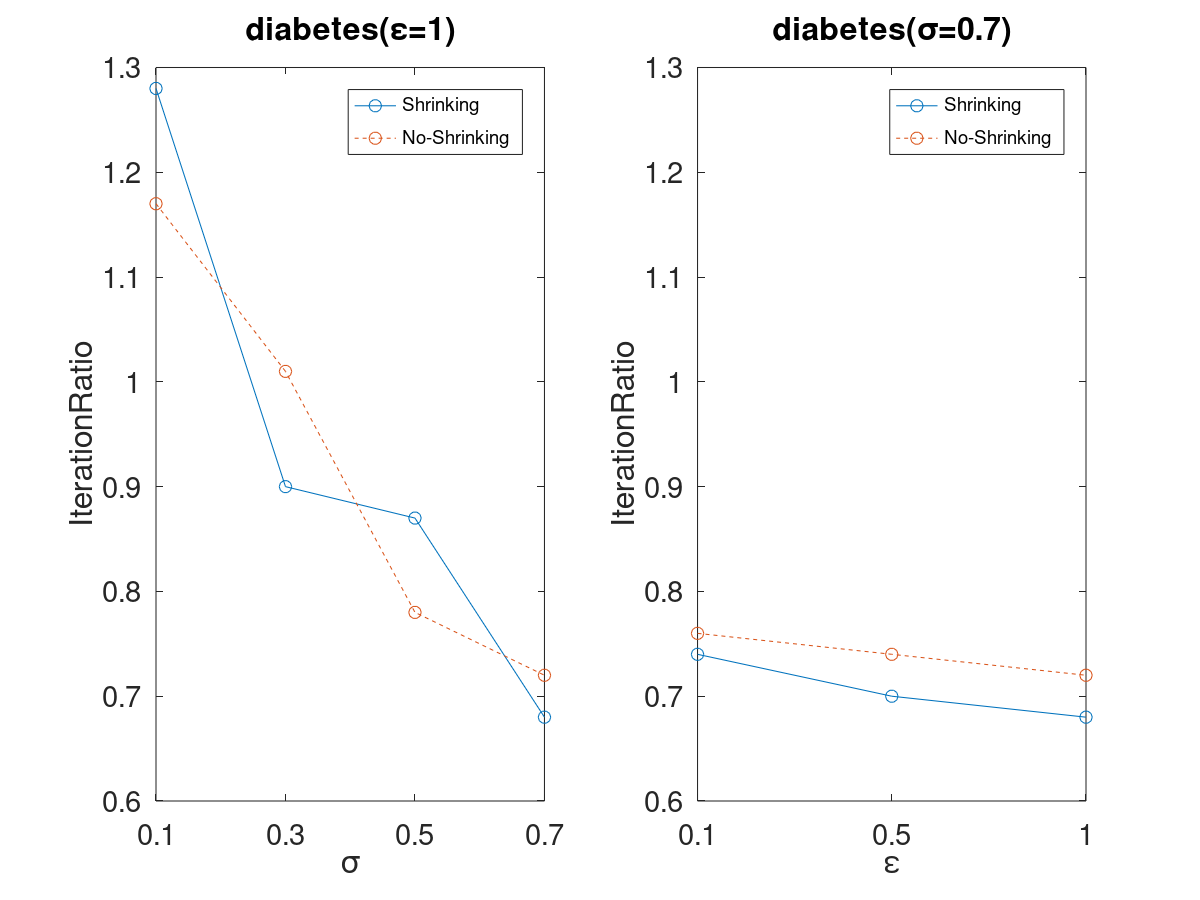

Supplement: Supplemental Information 1 [file peerj-cs-07-799-s001.zip › CODE/matlab/Figure/Figure10.png]

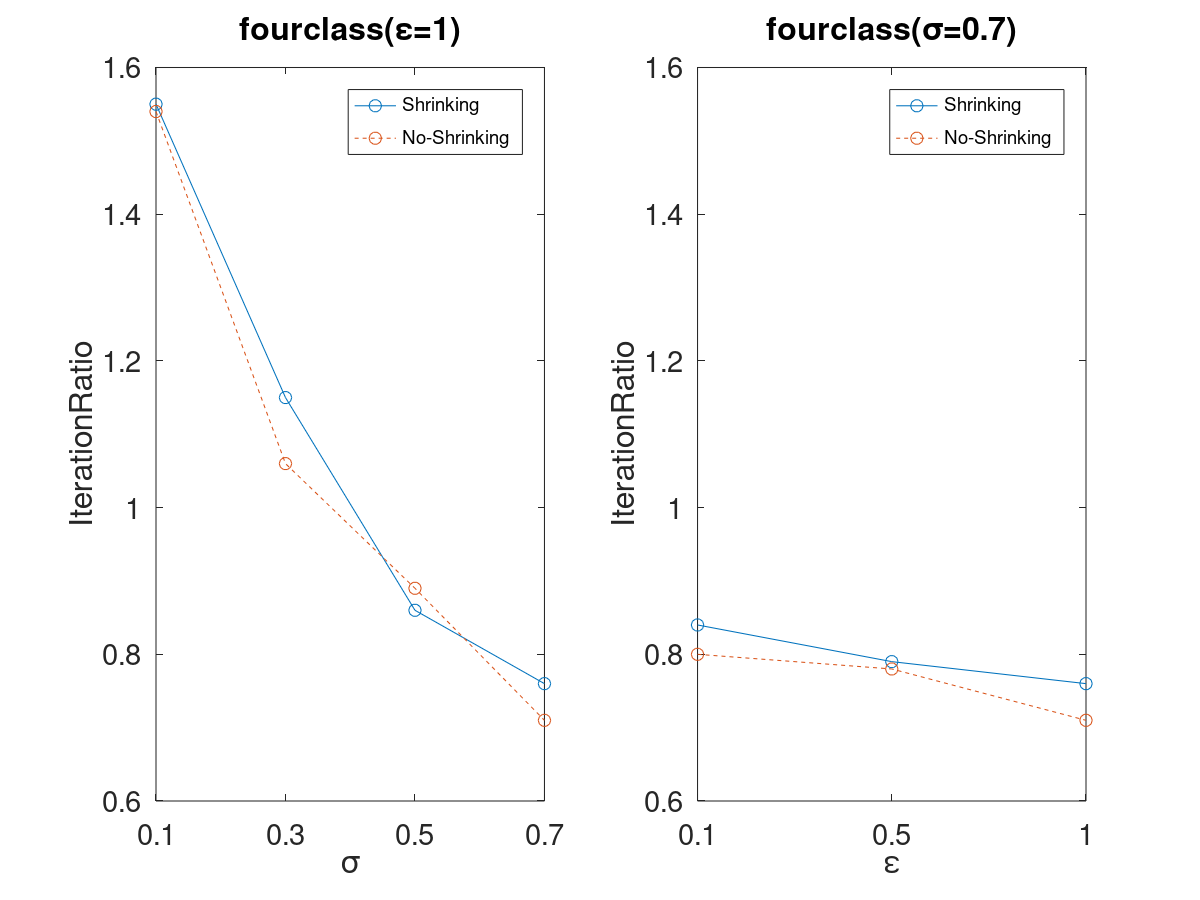

Supplement: Supplemental Information 1 [file peerj-cs-07-799-s001.zip › CODE/matlab/Figure/Figure11.png]

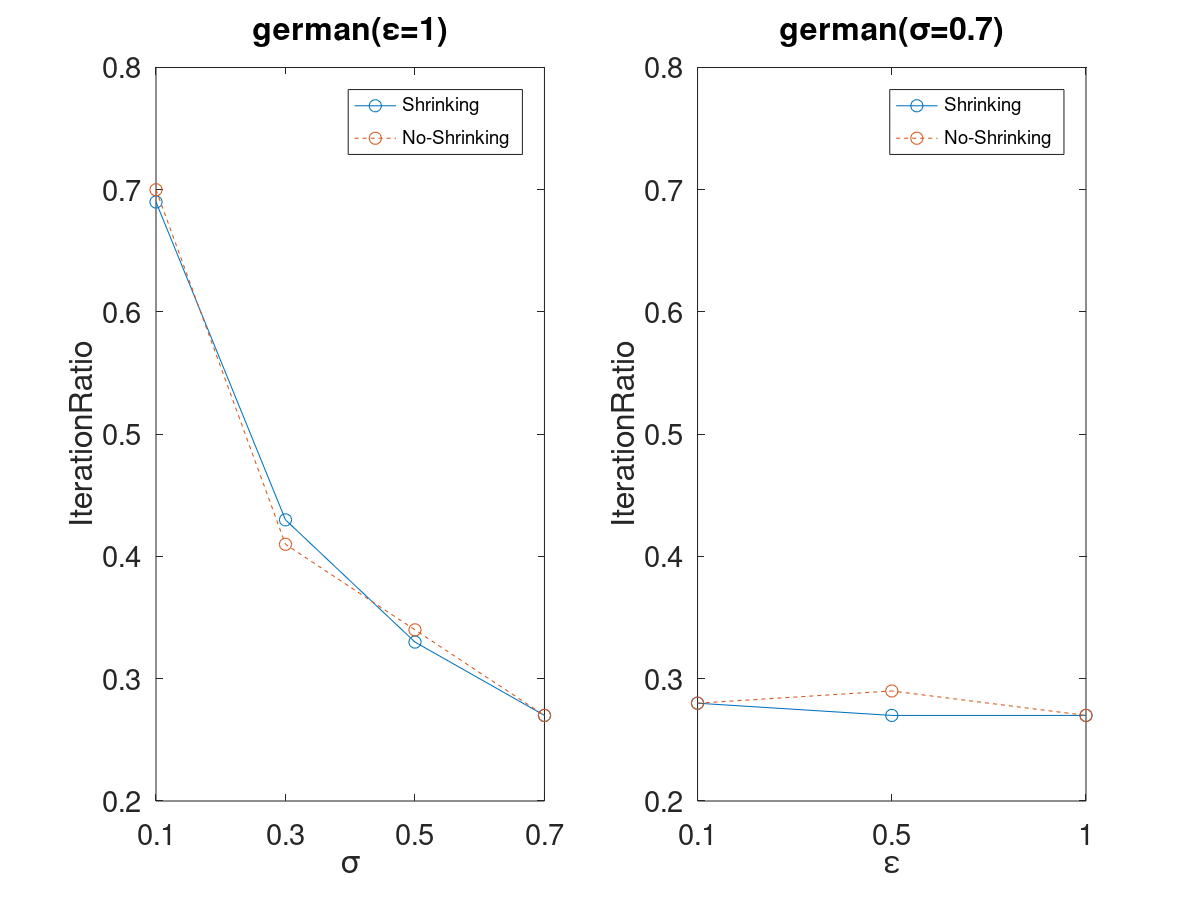

Supplement: Supplemental Information 1 [file peerj-cs-07-799-s001.zip › CODE/matlab/Figure/Figure12.png]

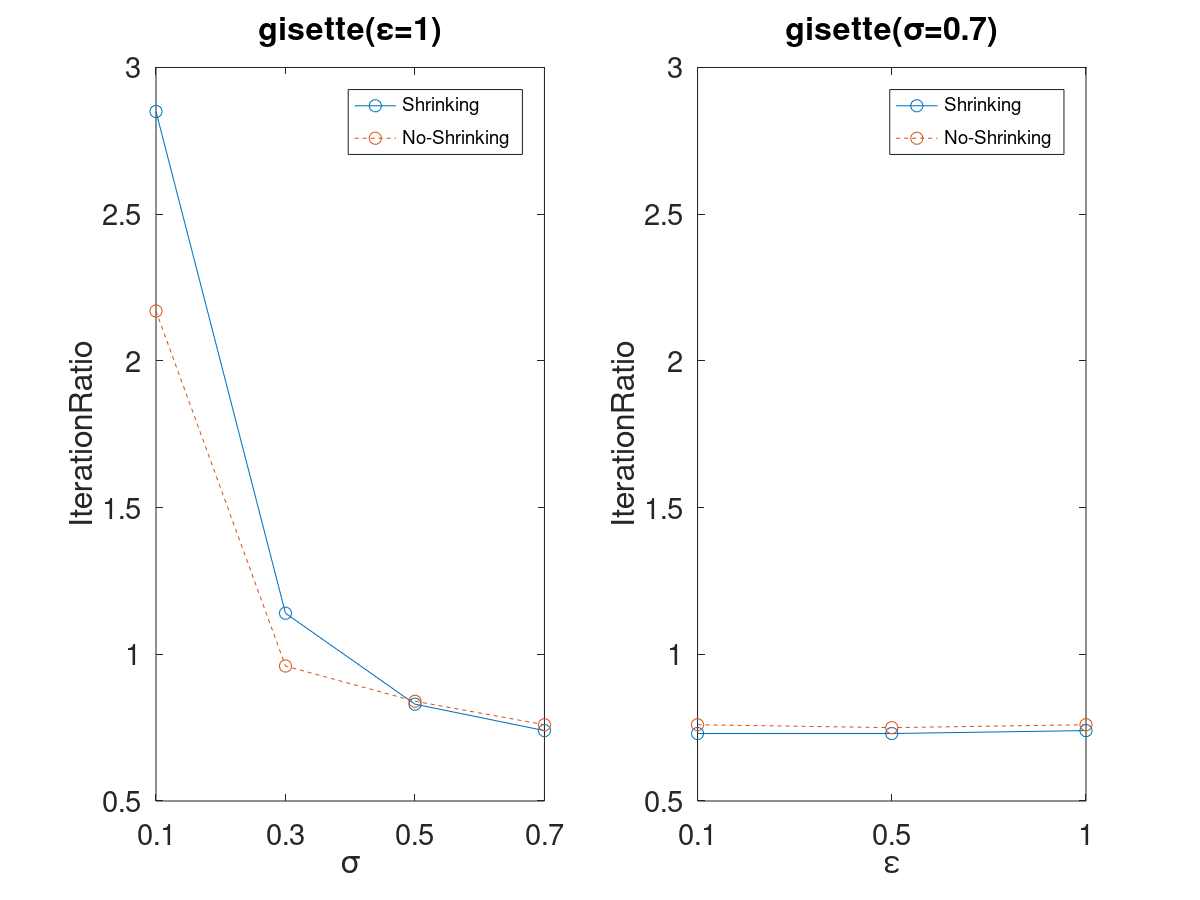

Supplement: Supplemental Information 1 [file peerj-cs-07-799-s001.zip › CODE/matlab/Figure/Figure13.png]

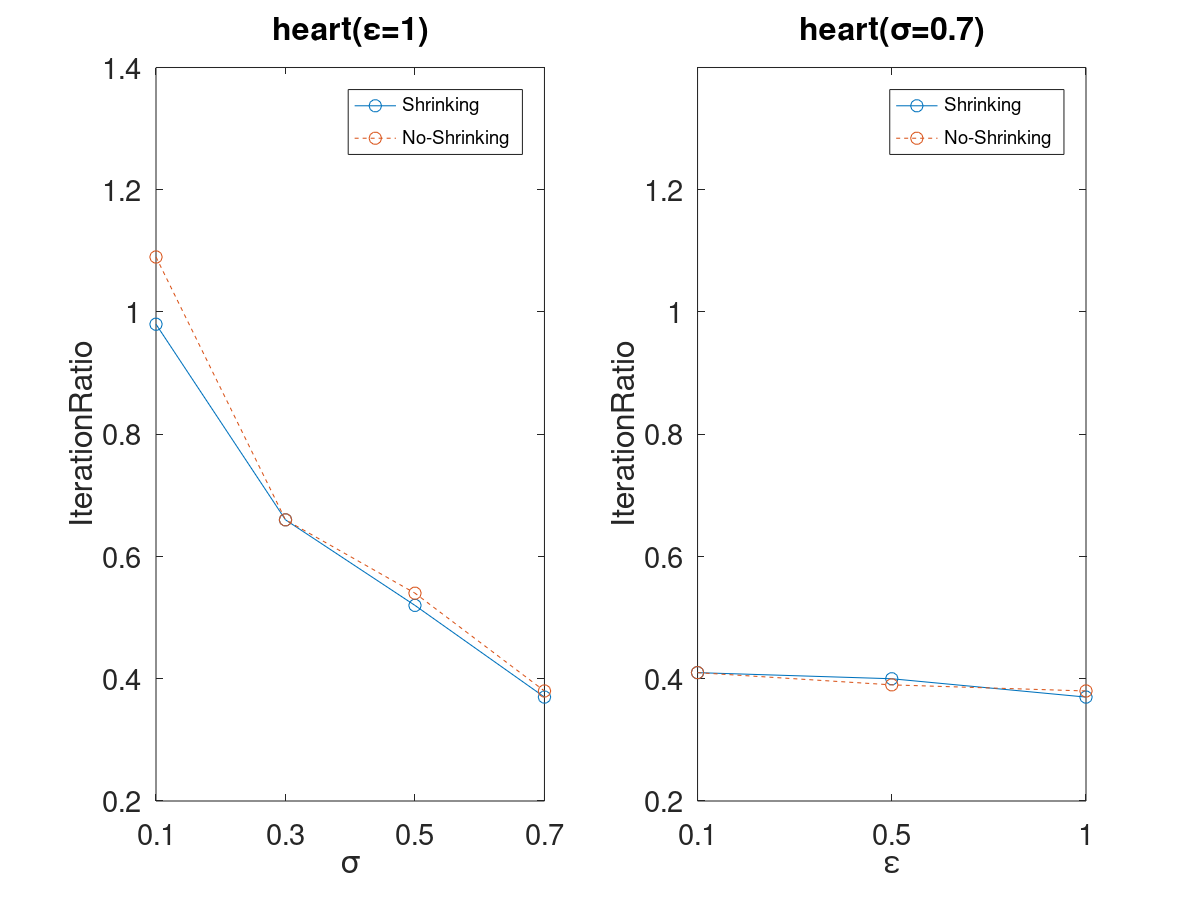

Supplement: Supplemental Information 1 [file peerj-cs-07-799-s001.zip › CODE/matlab/Figure/Figure14.png]

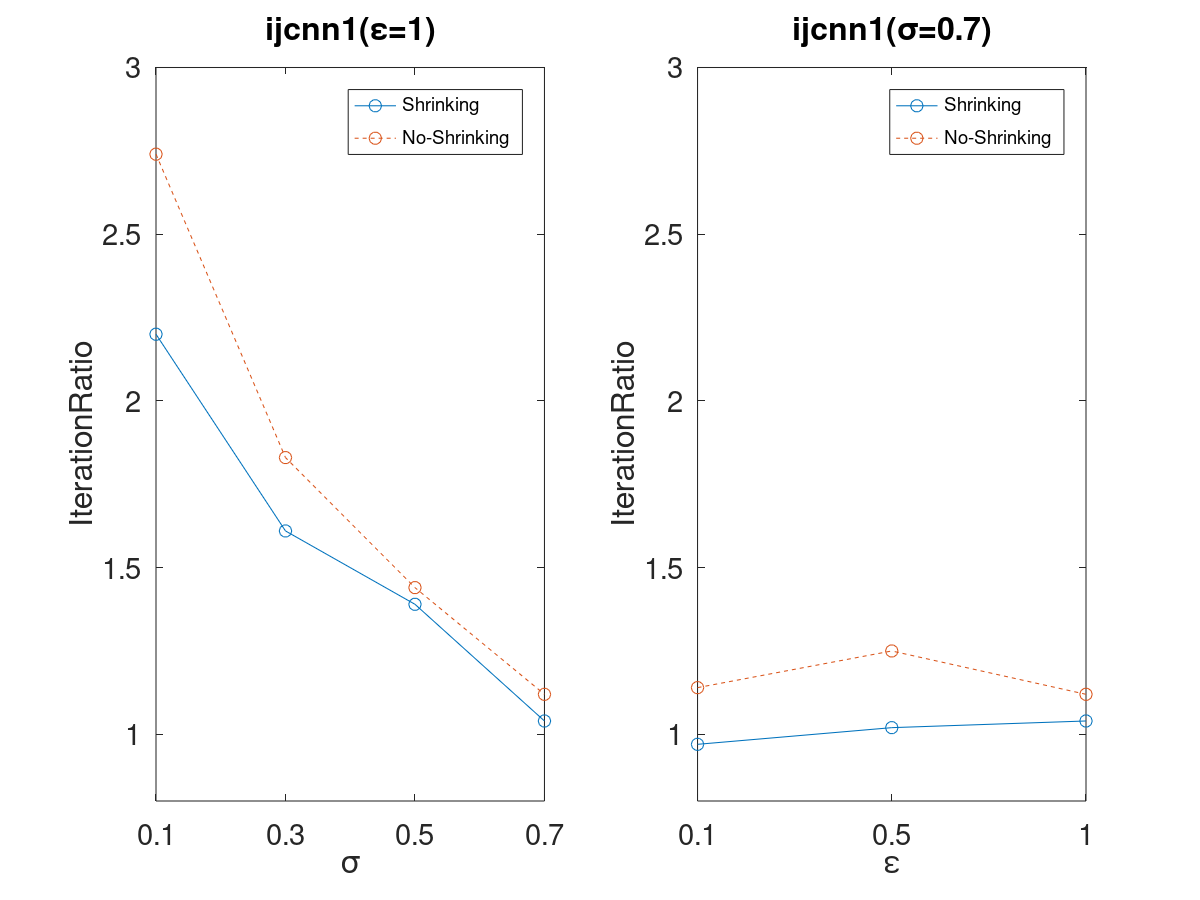

Supplement: Supplemental Information 1 [file peerj-cs-07-799-s001.zip › CODE/matlab/Figure/Figure15.png]

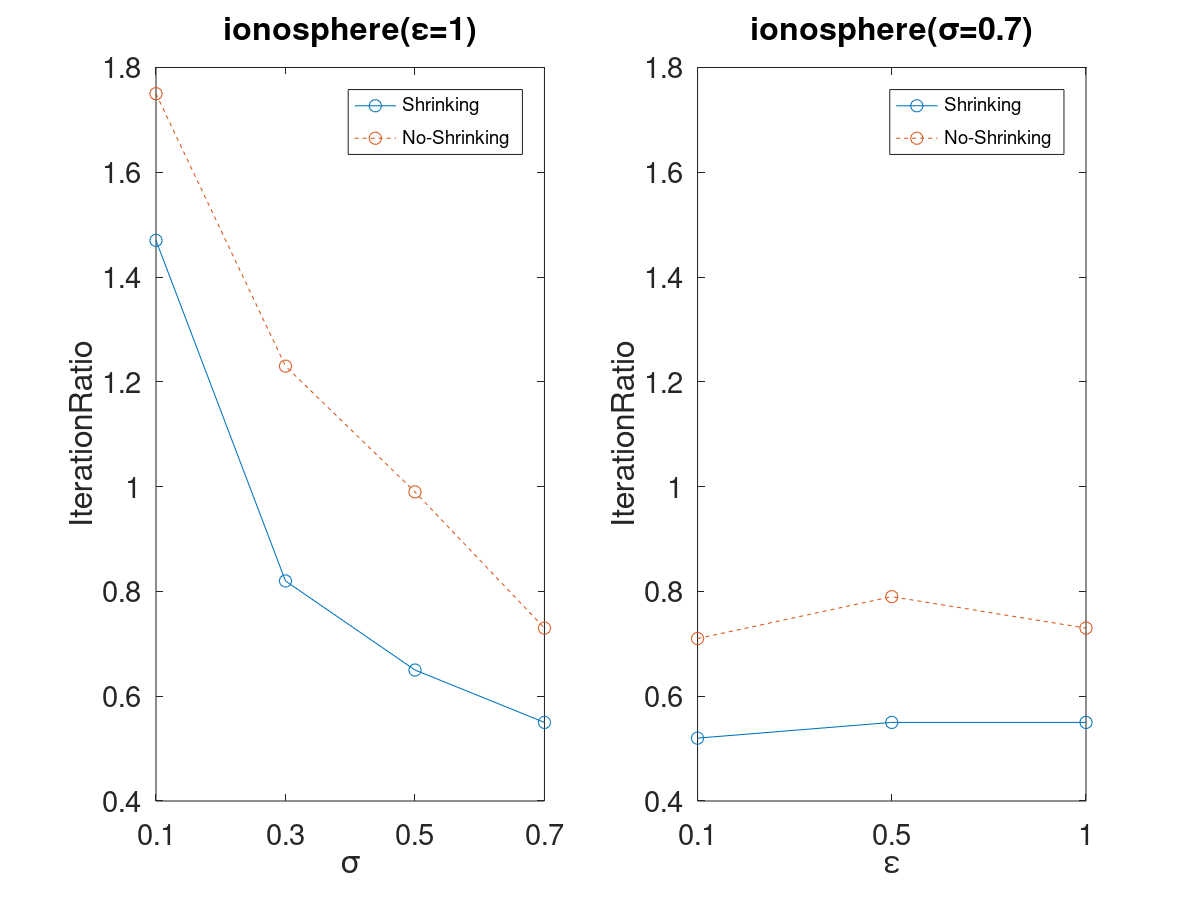

Supplement: Supplemental Information 1 [file peerj-cs-07-799-s001.zip › CODE/matlab/Figure/Figure16.png]

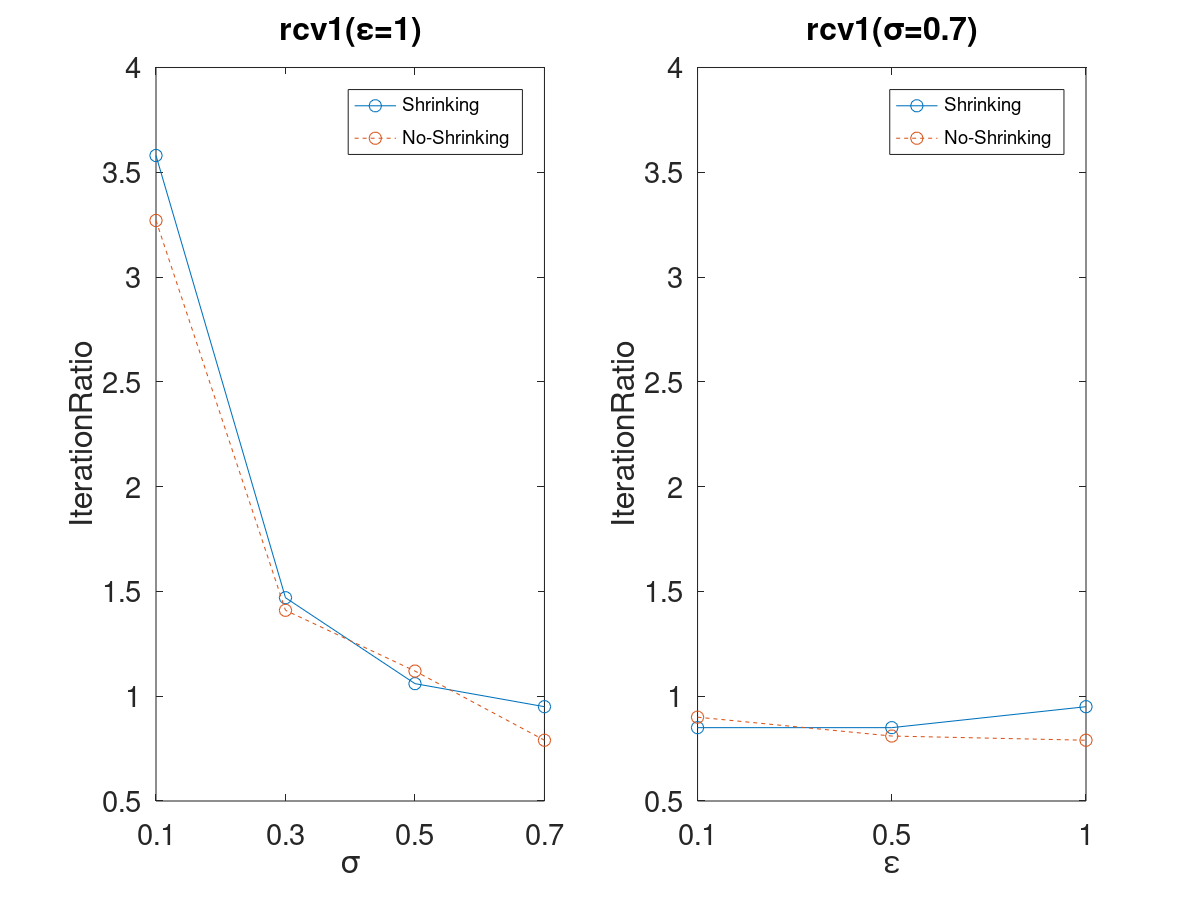

Supplement: Supplemental Information 1 [file peerj-cs-07-799-s001.zip › CODE/matlab/Figure/Figure17.png]

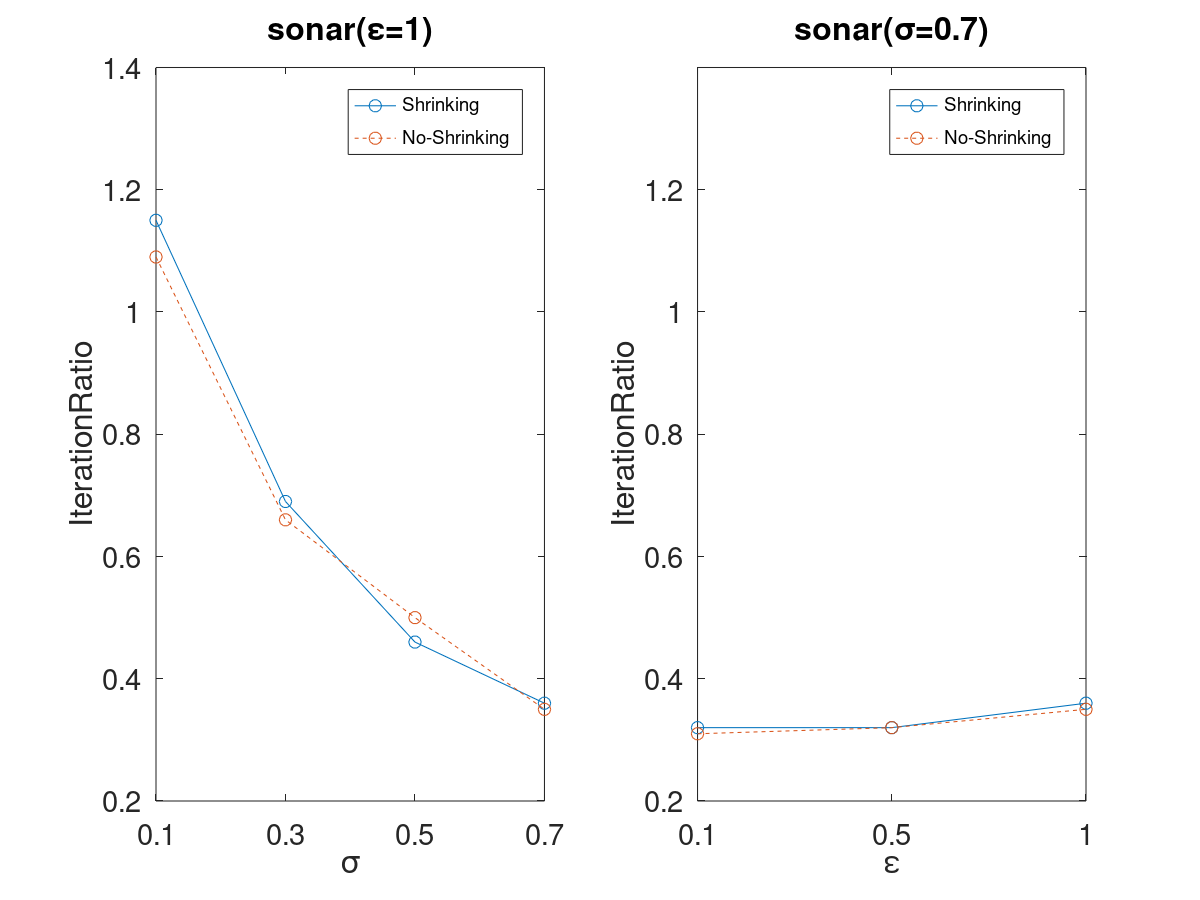

Supplement: Supplemental Information 1 [file peerj-cs-07-799-s001.zip › CODE/matlab/Figure/Figure18.png]

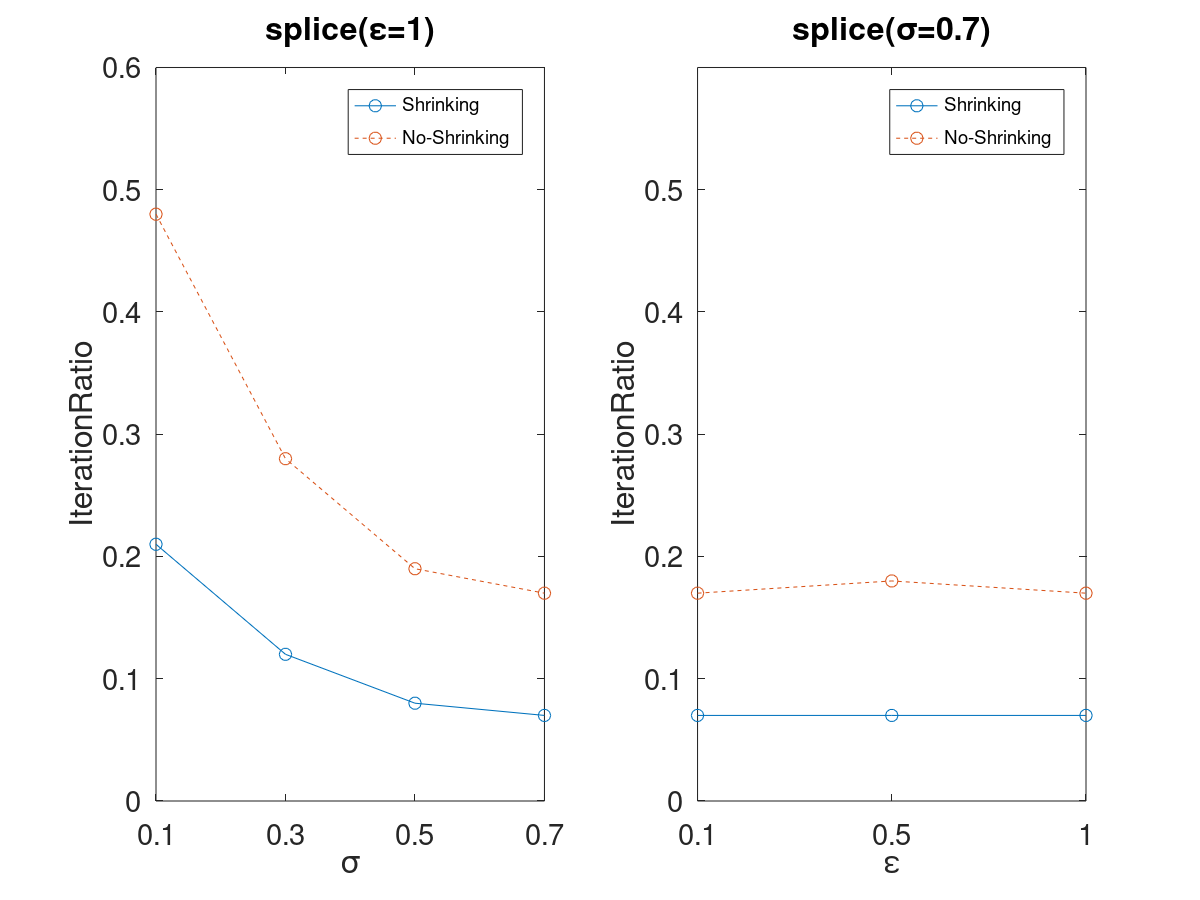

Supplement: Supplemental Information 1 [file peerj-cs-07-799-s001.zip › CODE/matlab/Figure/Figure19.png]

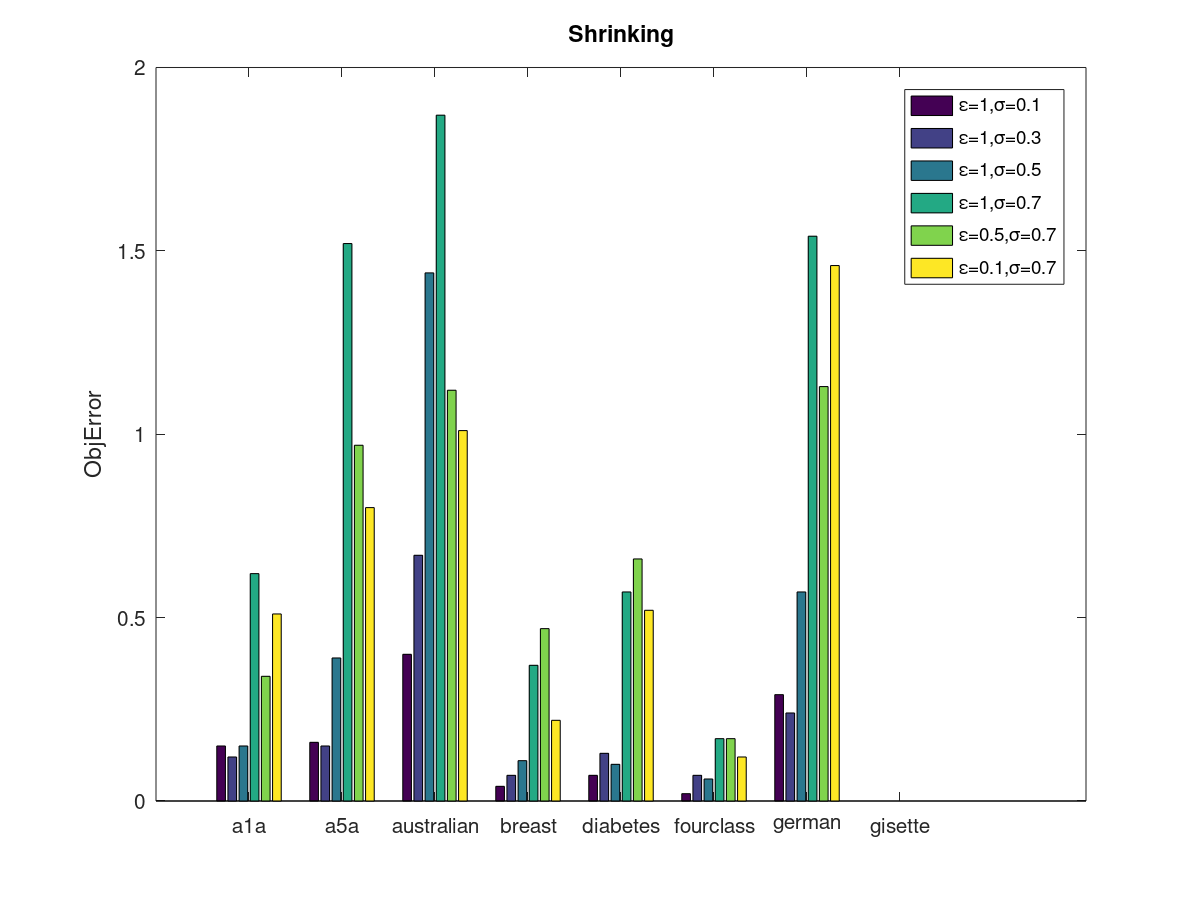

Supplement: Supplemental Information 1 [file peerj-cs-07-799-s001.zip › CODE/matlab/Figure/Figure2.png]

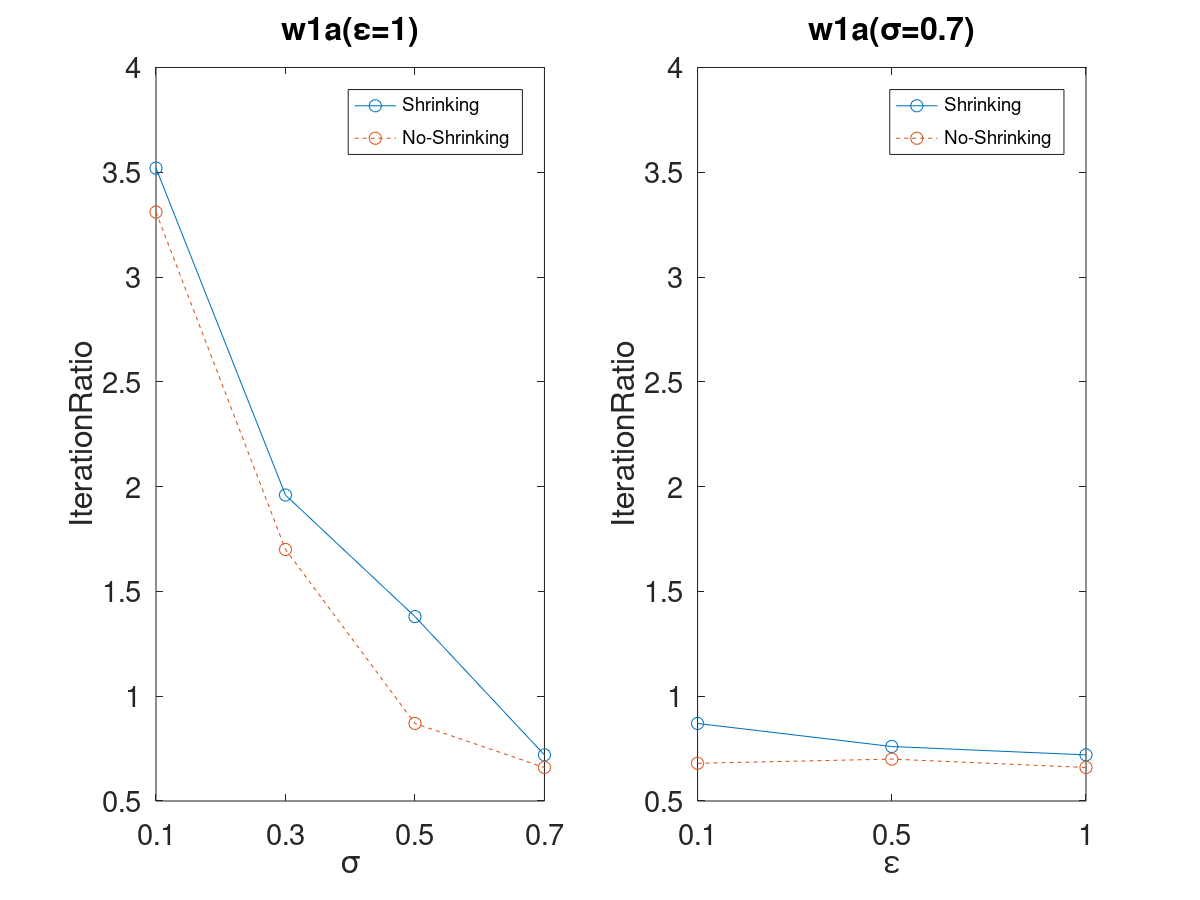

Supplement: Supplemental Information 1 [file peerj-cs-07-799-s001.zip › CODE/matlab/Figure/Figure20.png]

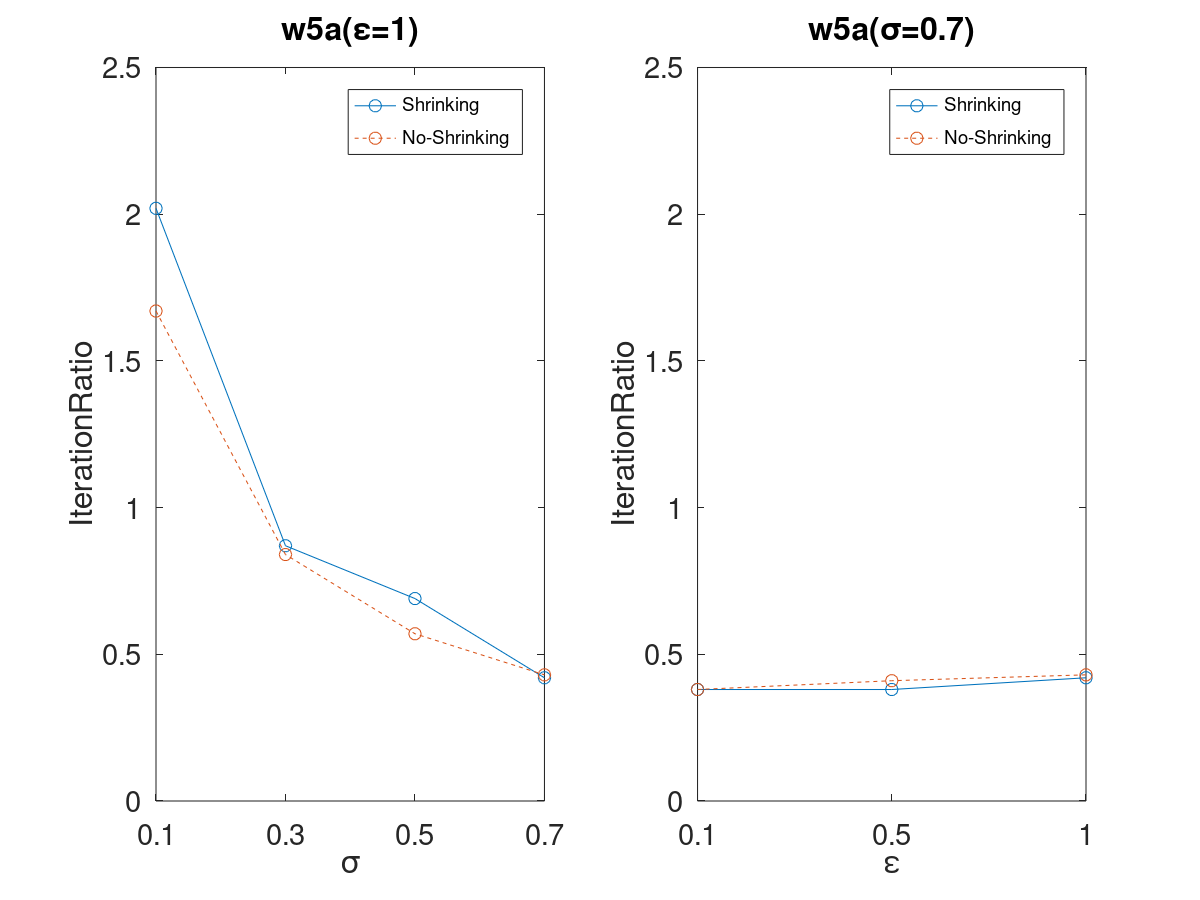

Supplement: Supplemental Information 1 [file peerj-cs-07-799-s001.zip › CODE/matlab/Figure/Figure21.png]

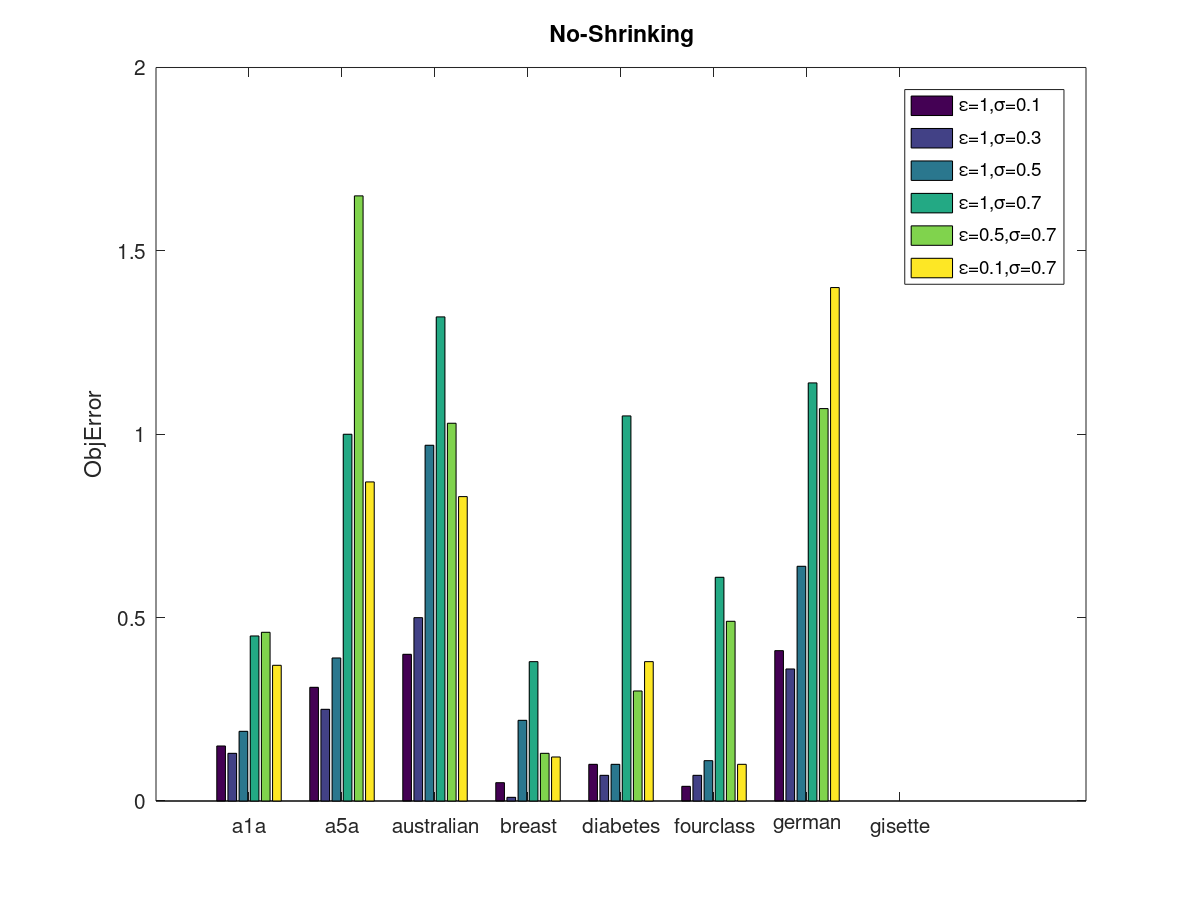

Supplement: Supplemental Information 1 [file peerj-cs-07-799-s001.zip › CODE/matlab/Figure/Figure3.png]

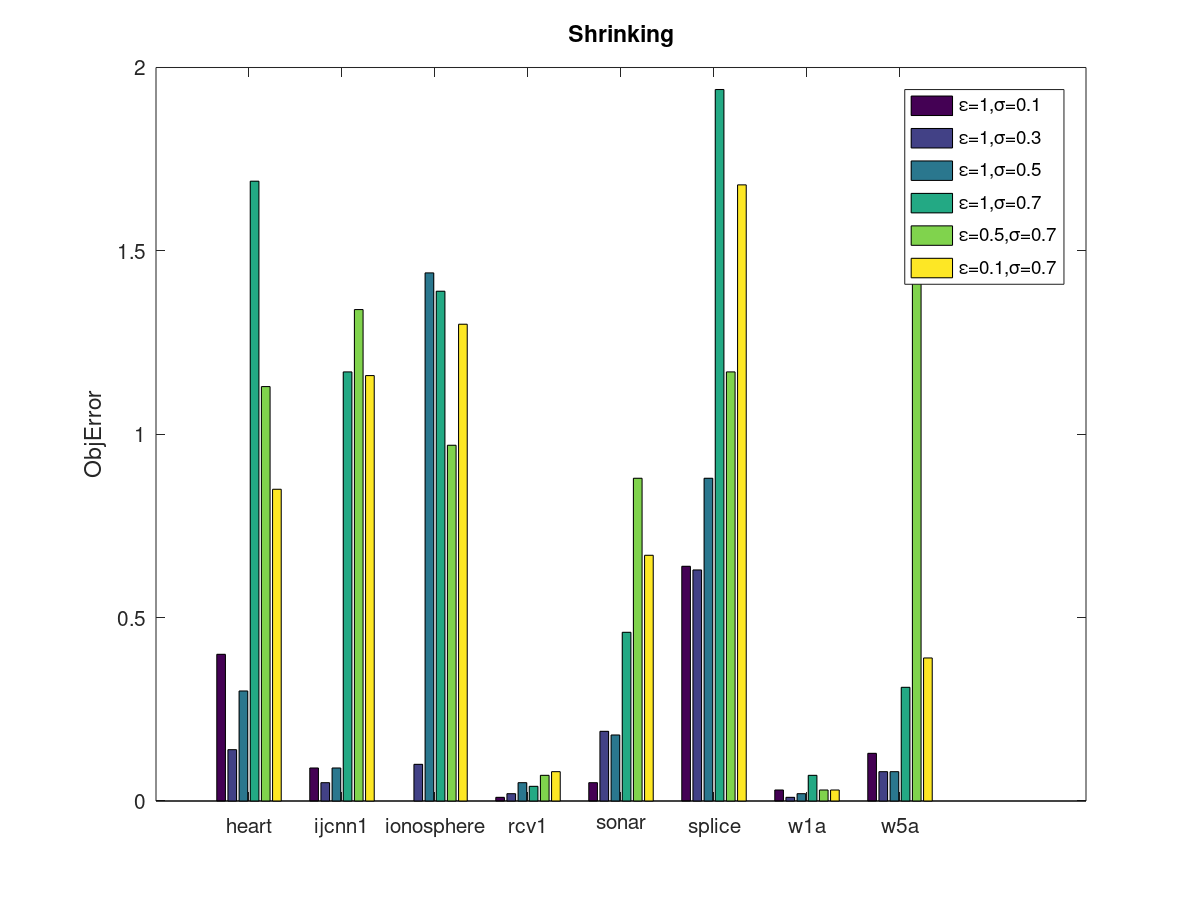

Supplement: Supplemental Information 1 [file peerj-cs-07-799-s001.zip › CODE/matlab/Figure/Figure4.png]

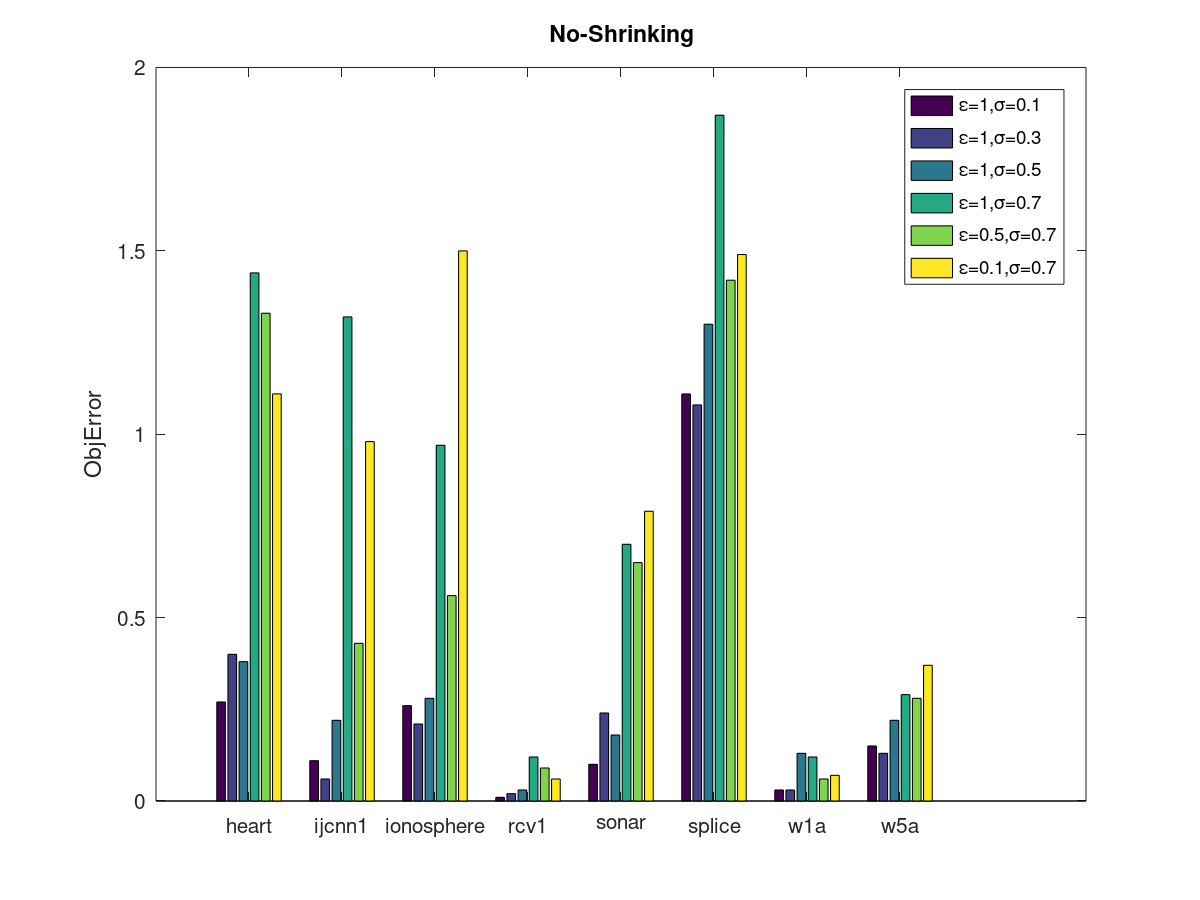

Supplement: Supplemental Information 1 [file peerj-cs-07-799-s001.zip › CODE/matlab/Figure/Figure5.png]

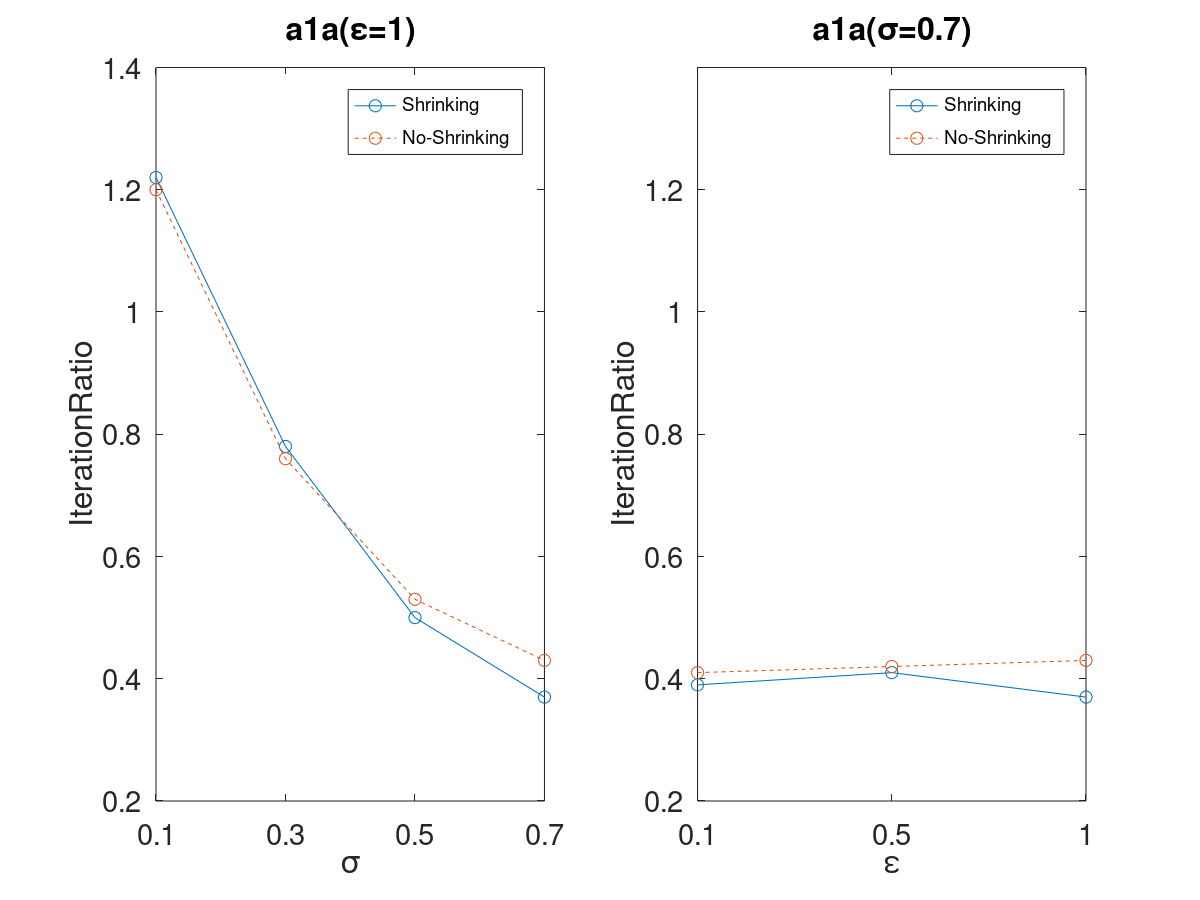

Supplement: Supplemental Information 1 [file peerj-cs-07-799-s001.zip › CODE/matlab/Figure/Figure6.png]

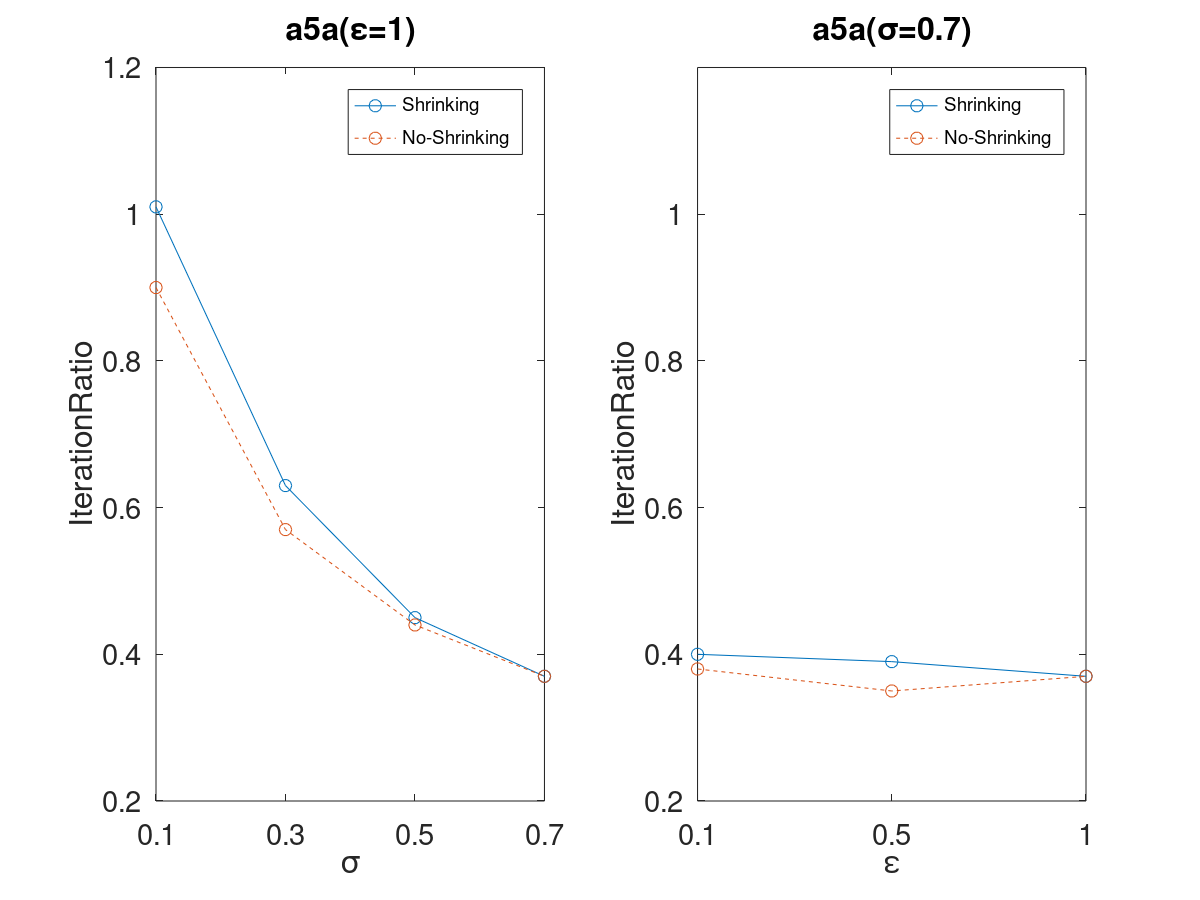

Supplement: Supplemental Information 1 [file peerj-cs-07-799-s001.zip › CODE/matlab/Figure/Figure7.png]

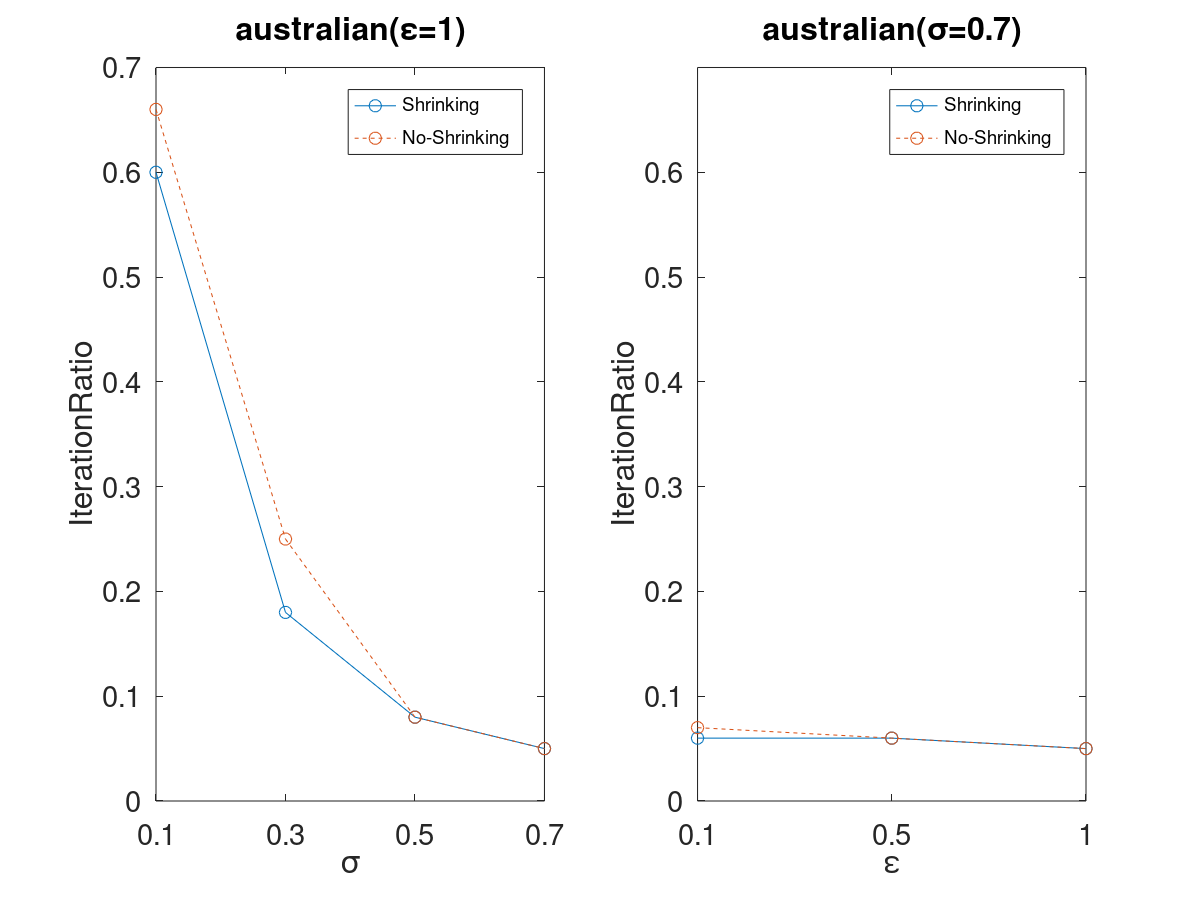

Supplement: Supplemental Information 1 [file peerj-cs-07-799-s001.zip › CODE/matlab/Figure/Figure8.png]

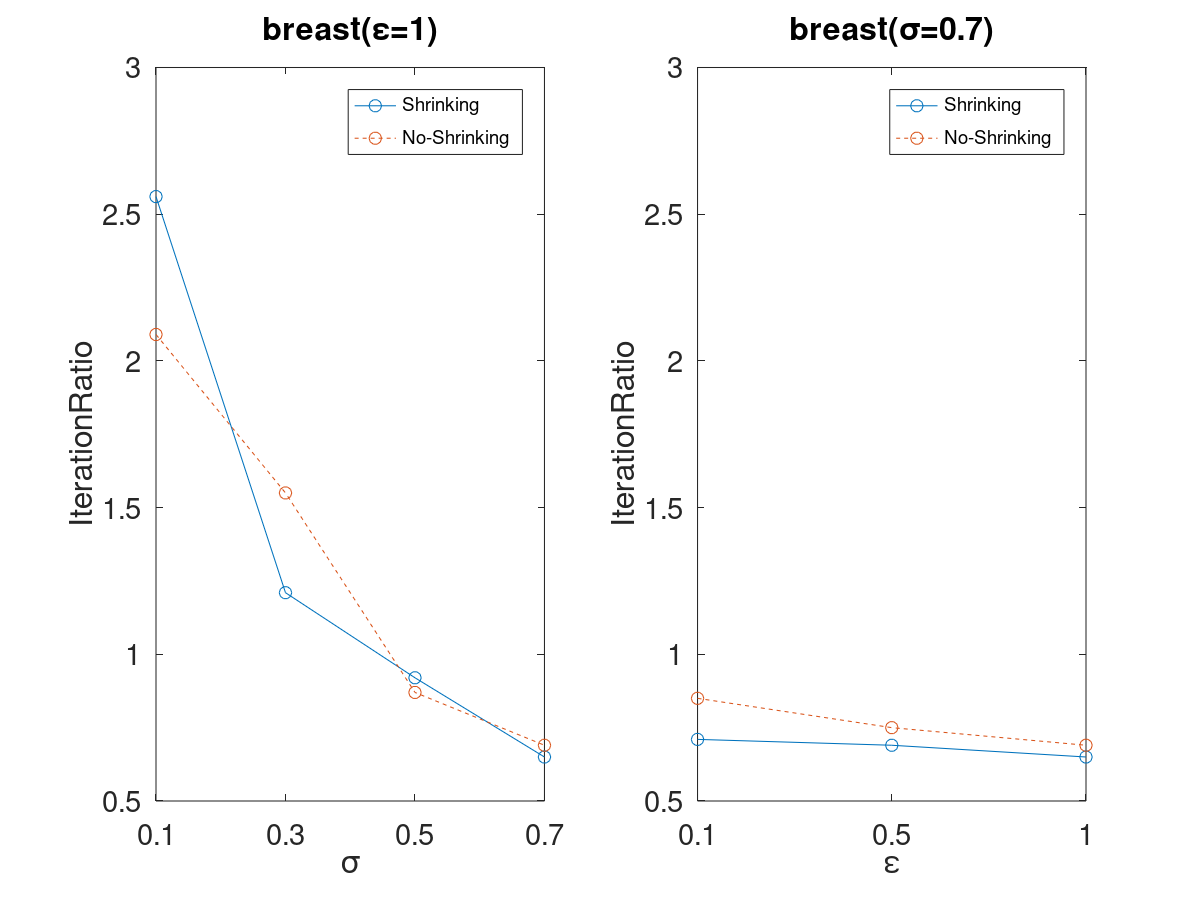

Supplement: Supplemental Information 1 [file peerj-cs-07-799-s001.zip › CODE/matlab/Figure/Figure9.png]
